# Kupffer Cell-derived IL6 Promotes Hepatocellular Carcinoma Metastasis Via the JAK1-ACAP4 Pathway

**DOI:** 10.7150/ijbs.97109

**Published:** 2025-01-01

**Authors:** Tao Li, Xiaoyu Song, Jiena Chen, Yuan Li, Jie Lin, Ping Li, Simiao Yu, Olanrewaju Ayodeji Durojaye, Fengrui Yang, Xing Liu, Jian Li, Shiyuan Cheng, Xuebiao Yao, Xia Ding

**Affiliations:** 1 School of Traditional Chinese Medicine, Beijing University of Chinese Medicine, Beijing, 100029, China.; 2 MOE Key Laboratory of Membraneless Organelle and Cellular Dynamics, Hefei National Laboratory for Physical Sciences at the Microscale, University of Science and Technology of China, Hefei, 230027, China.; 3 Dongzhimen Hospital, Beijing University of Chinese Medicine, Beijing, 100700, China.; 4 National Institute of Traditional Chinese Medicine Constitution and Preventive Treatment of Diseases, Beijing University of Chinese Medicine, Beijing, 100029, China.; 5 Department of Hepatobiliary, The Fifth Medical Center of PLA General Hospital, Beijing, 100039, China.; 6 Department of Neurology, Northwestern Malnati Brain Tumor Institute, The Robert H. Lurie Comprehensive Cancer Center, Northwestern University Feinberg School of Medicine, Chicago, IL, USA.

**Keywords:** Hepatocellular carcinoma, Tumor-associated macrophages, Kupffer cells, Janus kinase-1, ACAP4, Bufalin

## Abstract

Tumor-associated macrophages (TAMs), which differentiate from tissue-resident macrophages, are recognized for their ability to influence cancer progression and metastasis. However, the specific role of Kupffer cells (KCs), the intrinsic macrophages of the liver, in the progression of hepatocellular carcinoma (HCC) remains unclear. In this study, we describe a novel mechanism by which exosomes derived from HCC cells induce KCs to transition into TAMs, thereby facilitating the metastasis of HCC in an IL6-JAK1-ACAP4 axis-dependent manner. Mechanistically, the exosome-mediated domestication of KCs by hepatoma cells constitutes one of the primary sources of IL6 production in the HCC microenvironment. IL6 then activates JAK1 to phosphorylate its downstream effector ACAP4 at Tyr843, a novel phosphorylation site identified in this context, which in turn promotes ARF6-GTPase activity and hepatoma cell migration. Furthermore, we found that the levels of IL6, as well as the phosphorylation of JAK1 and ACAP4 at Tyr843, were significantly greater in tumor tissues from HCC patients than in adjacent tissues. These findings suggest that the IL6-JAK1-ACAP4 axis may be a promising therapeutic target for HCC. Importantly, we screened bufalin, an active ingredient derived from Venenum Bufonis, and discovered that it inhibits JAK1 and disrupts the IL6-induced phosphorylation of ACAP4. This inhibition not only impairs hepatoma cell migration but also prevents the metastasis of HCC. These findings demonstrate the interplay between hepatoma cells and KCs through the IL6-JAK1-ACAP4 axis, thereby promoting HCC metastasis, and reveal the therapeutic potential of bufalin for the treatment of HCC through JAK1 inhibition.

## Introduction

Hepatocellular carcinoma (HCC) is the most common type of primary liver cancer (90%) in East Asia and Africa, and has the highest incidence 1. This malignancy is notorious for its chronic nature, rapid tumor progression, and frequent recurrence rates after surgical resection; thus, it is difficult to treat and has a poor prognosis 2. Tumor-associated macrophages (TAMs) are macrophages that reside within the tumor microenvironment (TME) and are linked with tumor progression and metastasis because of their protumoral activities. Tumor cells crosstalk with tumor-infiltrating macrophages by secreting exosomes to promote macrophage differentiation into TAMs and thereby secrete various inflammatory cytokines, chemokines, and matrix metalloproteinases in the TME to establish a tumor-promoting and immunosuppressive microenvironment. Kupffer cells (KCs) are macrophages specific to the liver tissue and are important members of the innate immune system 3. KCs reside within the liver sinusoidal vascular space, predominantly in the periportal area, and respond directly to acute liver injury and bacterial and viral infection 4. KCs have also been implicated in the pathogenesis and progression of HCC through their ability to undergo phenotypic changes and secrete cytokines 5. While prior research has focused on the interactions between hepatoma cells and circulating monocytic macrophages, revealing that these macrophages adopt an M2-like phenotype in response to the local microenvironment 6,7, the role of KCs as liver-resident macrophages in the advancement of HCC remains inadequately explored.

Interleukin-6 (IL6) is a pleiotropic four-helical cytokine of 184 amino acids that is synthesized and secreted by fibroblasts, monocytes, T cells, and macrophages. In the liver, IL6 is an important mediator of the acute-phase immunological response, infection defense, liver regeneration, and hepatic metabolism. In addition, IL6 is also crucial for the progression and metastasis of HCC through the activation of Janus kinase-1 (JAK1) and its downstream signal transducer and activator of transcription (STAT), which directly stimulate the proliferation, migration, and invasion of tumor cells. The IL6 signaling pathway is initiated by its binding to the IL6 receptor (IL6R) and subsequent recruitment of glycoprotein 130 (GP130), leading to the activation of the JAK family of kinases. JAK1, along with JAK2, JAK3, and TYK2, are members of this family and are implicated in both inflammatory disorders and cancer 8. To date, several JAK inhibitors have received approval for the treatment of hematological malignancies and inflammatory conditions 9,10. However, despite these advancements, no JAK inhibitor has yet been clinically validated for the treatment of HCC.

Tumor metastasis have been linked with cancer cell migration and invasion. During cell migration, actin assembly and plasma membrane dynamics, which are regulated by the actin cytoskeleton and associated motor proteins are required for protrusive activities at the leading edges of the migrating cells 11. Some small GTPases, such as ADP-ribosylation factor 6 (ARF6), are widely expressed in mammalian cells and mainly regulate the functions of membrane trafficking and actin remodeling by cycling between inactive GDP-binding conformations and active GTP-binding conformations 12. Our previous study revealed that Rho kinase-mediated phosphorylation of cytoskeleton-membrane linker proteins promotes HCC metastasis 13. ACAP4, an ARF6-GTPase-activating protein, selectively binds to ARF6 and catalyzes GTP hydrolysis to regulate membrane cytoskeletal dynamics 14, and the phosphorylation of ACAP4 at Tyr733 orchestrates EGF-stimulated integrin β1 recycling during cell migration 15,16. Our recent study demonstrated that the acetylation of ACAP4 at Lys311 is essential for CCL18-induced breast cancer cell migration and invasion via the modulation of ARF6-GTP near the plasma membrane 17. However, the involvement of the ACAP4‒ARF6 axis in interleukin-6 (IL6)-mediated hepatoma cell migration and its precise regulatory role in HCC metastasis remain to be elucidated.

Here, we found that, compared with control counterparts, IL6 was significantly upregulated in the serum and tumor tissues of HCC patients, with a negative correlation with the prognosis of patients. KCs are a significant source of IL6 production within the HCC microenvironment. The interplay between KCs and exosomes derived from HCC induced KCs to differentiate into TAMs, which secreted IL6 to activate JAK1 in hepatoma cells. JAK1 phosphorylated ACAP4 at Tyr843, a previously unreported phosphorylation site on the ACAP4 protein, thereby enhancing HCC cell migration by increasing ARF6-GTPase activity. Furthermore, we assessed the clinical significance of ACAP4 phosphorylation at Tyr843 (p-ACAP4^Y843^) as a biomarker of HCC metastasis through the analysis of tumor tissue samples from patients. In the final phase of our study, we screened natural product derivatives for active ingredients with potential against HCC and examined whether bufalin could target JAK1 to inhibit hepatoma cell migration and impede the metastasis of HCC.

## Results

### IL6 is upregulated in the serum and tumor tissue of HCC patients and is correlated with an unfavorable prognosis

**Figure 1:**
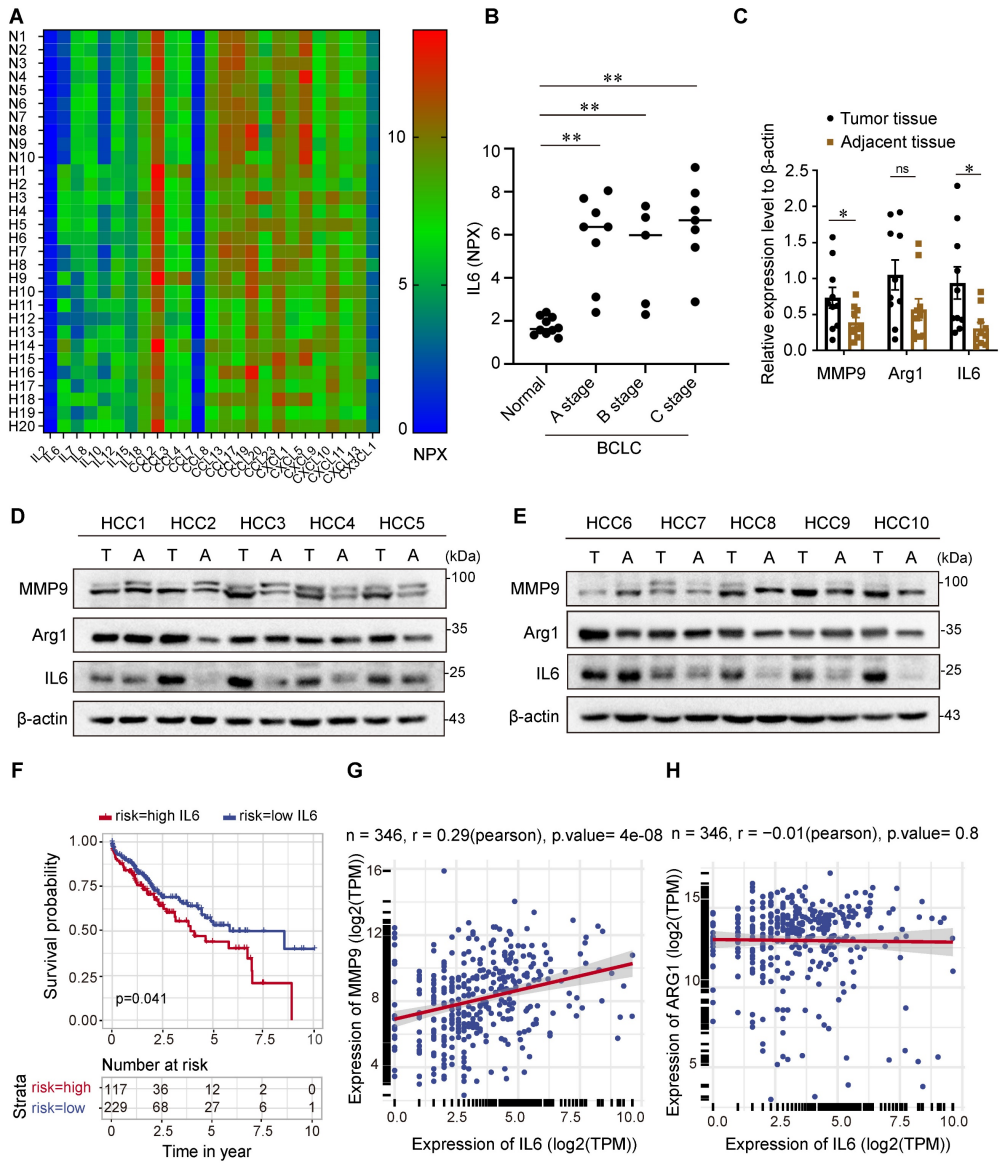
** IL6 is upregulated in the serum and tumor tissue of HCC patients and is correlated with an unfavorable prognosis. (A)** Heatmap illustrating the differential expression of 25 serum cytokines in 10 healthy individuals and 20 HCC patients as determined by the Olink high-throughput proteomics assay. **(B)** Statistical analysis revealed that patients with HCC at stages A, B, and C of the BCLC classification had higher levels of the IL6 protein in the serum than did the controls. The data are presented as the average ± SEM. **p < 0.01. **(C)** Statistical analysis of (D) revealed high protein expression of IL6 and MMP9 in HCC tissues compared with adjacent tissues (n=10). The data are presented as the average ± SEM. *p < 0.05, ^ns^ *p* > 0.05. **(D, E)** Immunoblotting (IB) analysis of the IL6, MMP9, and Arg1 protein levels in HCC tissues and adjacent tissues. **(F)** Kaplan‒Meier analysis of the associations between the IL6 level and overall survival of HCC patients (*p*<0.05). **(G, H)** Correlation analysis revealed a positive correlation between the IL6 level and the MMP9 level in the TCGA HCC dataset (*p*<0.01).

To determine the cytokines indicative of HCC risk, 92 immuno-oncology proteins from the serum of HCC patients and normal participants were analyzed using the OLINK high-throughput proteomics assay. Then, 25 serum cytokines were screened out and presented in a heatmap (Figure 1A and Table S1), of which IL6 and IL10 were upregulated in the serum of HCC patients compared with that of normal participants. IL-10 is widely accepted as an immunosuppressive cytokine and has paradoxical effects on the pathogenesis and development of HCC 18,19.

IL6, however, is thought to be secreted by TAMs in the TME and implicated in the pathogenesis and development of HCC 20. Therefore, we focused on the role of IL6 in tumor progression and measured the serum levels of IL6 in patients with HCC across stages A, B, and C of the Barcelona Clinic Liver Cancer (BCLC) classification. As shown in Figure 1B, the serum levels of IL6 were elevated in patients with BCLC stage A, B, and C HCC compared with healthy individuals. In addition, we analyzed the protein levels of IL6 and markers of TAMs, including matrix metalloproteinase 9 (MMP9) and arginase 1 (Arg1), in HCC cancer tissues and adjacent tissues (Figure 1C-1E). Our results revealed higher levels of IL6 and MMP9 in HCC tissues than in adjacent tissues. However, there was no significant difference in the expression of Arg1 between cancer tissues and adjacent tissues.

We further explored the relationship between the level of IL6 and HCC prognosis via data from the TCGA database. The Kaplan‒Meier survival curve in Figure 1F indeed demonstrated poor overall survival among patients with high expression of IL6, which was statistically significant (*p*<0.05). Additionally, Pearson's correlation analysis revealed that the expression level of IL6 was positively correlated with the expression of MMP9 (*p*<0.01, Figure 1G-1H).

### Kupffer cells are important sources of IL6 in the microenvironment of HCC

**Figure 2:**
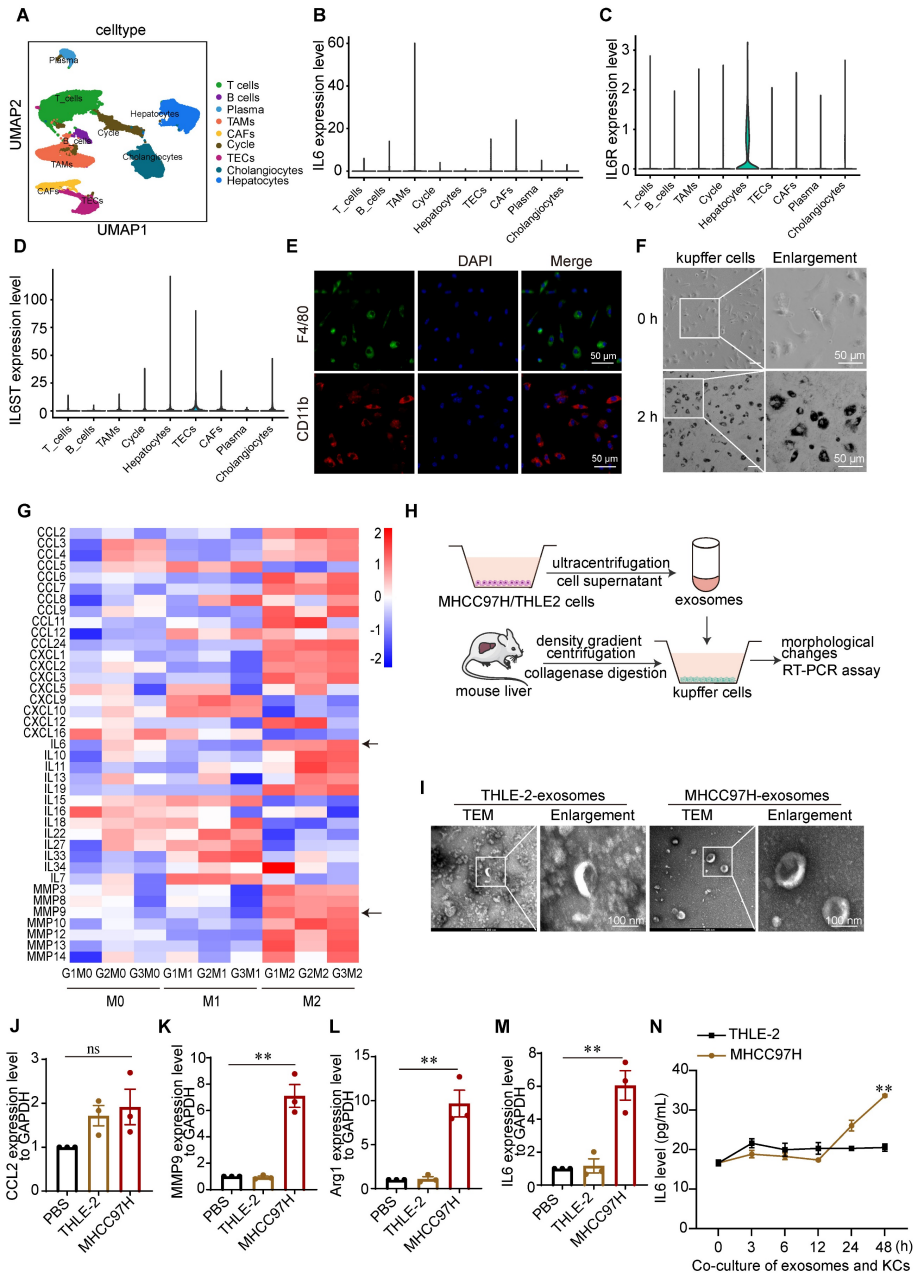
** Kupffer cells are important sources of IL6 in the microenvironment of HCC. (A)** The t-distributed stochastic neighbor embedding plot of all the single cells, with each color coding for major cell clusters derived from HCC samples on the basis of single-cell RNA-seq analysis of the GEO dataset (GSE151530). **(B-D)** UMAP plot of IL6, IL6R, and IL6ST expression in different cell clusters. **(E)** Immunofluorescence (IF) staining was used to measure F4/80 and CD11b expression in KCs derived from mouse livers (scale bar, 50 μm). **(F)** Ink phagocytic experiment of KCs at 0 and 2 h (scale bar, 50 μm). **(G)** Heatmap of the transcription levels of chemokines, inflammatory cytokines, and matrix metalloproteinases in M1/M2-linked macrophages, as determined via RNA-seq. **(H)** Schematic diagram of the coculture of mouse liver KCs with exosomes derived from MHCC97H or THLE-2 cells. **(I)** Electron microscopy analysis of exosomes derived from THLE-2 and MHCC97H cells (scale bar, 100 nm). **(J-M)** RT-PCR analysis of the expression of M1 marker CCL2 and TAMs markers, including MMP9, Arg-1, and IL6, in KCs after the coculture of KCs with exosomes derived from MHCC97H or THLE-2 cells. **p < 0.01, ^ns^ *p* > 0.05. **(N)** ELISA analysis of the protein level of IL6 in the supernatants of KCs cocultured with exosomes derived from MHCC97H cells and THLE-2 cells. The data are presented as the average ± SEM. **p < 0.01.

It has been reported that IL6 is synthesized by monocytes, macrophages, and T cells 21. To determine which immune cells differentiated into TAMs in the HCC TME and contributed to IL6 secretion, we analyzed the single-cell (sc) RNA-seq data of 46 HCC patients downloaded from the GEO database (GSE151530). Cell clusters were annotated on the basis of the expression of curated known cell markers on the UMAP plot (Figure 2A), and the levels of IL6, IL6R and IL6ST in different cell clusters were analyzed. IL6R and IL6ST exhibited elevated expression levels across most cell types within the TME, encompassing hepatocytes, cholangiocytes, and TAMs. In contrast, IL6 expression was predominantly elevated in TAMs (Figure 2B-D).

Previous studies on the interaction between cancer cells and TAMs in liver cancer have focused mainly on bone marrow-derived mononuclear macrophages and liver cancer cells 6. However, liver-resident KCs are the major source of macrophages in the hepatic sinusoid and play important roles in the maintenance of liver homeostasis as well as the incidence and progression of HCC. We therefore isolated mouse liver KCs via percoll density gradient centrifugation (Figure S1A). using immunofluorescence (IF) staining, F4/80 and CD11b expression were detected in most of the isolated KCs (Figure 2E). Additionally, the phagocytic activity of KCs was verified by an ink phagocytic assay (Figure 2F). These results indicated that the isolated mouse KCs were viable.

Since TAMs and their M2-like alternative activation significantly contribute to the development of HCC, we used LPS + IFN-γ and IL4 +IL13 to induce M1/M2 activation of KCs, respectively 22. As expected, marked changes in cell shape after macrophages polarized toward different phenotypes were observed. Consistent with the findings of a previous study 22, M2-like cells exhibited an elongated shape, whereas M1-like cells exhibited a round and pancake-like shape (Figures S1B-1C). To determine the transcription profiles of chemokines, inflammatory cytokines, and matrix metalloproteinases in M1/M2-linked macrophages, we performed RNA-seq analyses (Figure S1D-1F). IL6 and MMP9 were highly expressed in M2-like macrophages, the polarization type most closely related to TAMs (Figure 2G).

TAMs generated from exosomes derived from hepatoma cells produce inflammatory cytokines, angiogenic molecules, and proteases, facilitate angiogenesis and matrix breakdown, and promote tumor progression and metastasis. Therefore, exosomes derived from the culture medium of HCC-MHCC97H cells and normal human liver THLE-2 cells were extracted and cocultured with KCs to simulate the TME (Figure 2H). The marker proteins of the exosomes were validated by western blot analyses (Figure S2A-2B). The morphologies and sizes of the exosomes were further determined by transmission electron microscopy (TEM) and nanoparticle tracking analysis (NTA). The mean diameter of the extracted exosomes from MHCC97H cells was 123.6 nm, and the mean diameter of the extracted exosomes from THLE-2 cells was 103.2 nm (Figure S2C-2D). TEM microscopy revealed cup-shaped and disc-like exosomes (Figure 2I). IF staining revealed that exosomes labeled with PKH26 were taken up by KCs after coculture for 2 h (Figures S2E-2F). To further confirm that KCs are induced by tumor cell-derived exosomes and secrete IL6, we determined the expression of IL6 and markers of M1-like macrophages and TAMs by RT-PCR. Our findings indicate that there was no significant difference in the expression of CCL2, whereas IL6, MMP9, and Arg1 were upregulated in KCs following coculture with exosomes derived from MHCC97H cells (Figure 2J-2M). Additionally, ELISA analysis revealed that the level of the IL6 protein in the supernatants of KCs cocultured with exosomes derived from MHCC97H cells was greater than that in the supernatants of KCs cocultured with exosomes from THLE-2 cells (Figure 2N). To further investigate the underlying mechanisms, we conducted mass spectrometry analysis on exosomes and found that SLC7A11 was the most abundant protein in exosomes originating from MHCC97H cells, in comparison to those derived from THLE-2 cells (Figure S3A-D).

Notably, previous studies have demonstrated that SLC7A11 is upregulated in M2-like macrophages and TAMs 23,24. SLC7A11 promotes HCC metastasis by increasing programmed death ligand 1 and colony-stimulating factor 1, and its high expression is linked to poor tumor differentiation and an advanced tumor-node-metastasis stage 25. In light of the previously mentioned findings, we did not further examine the effect of SLC7A11 in exosomes on KCs in this study. Instead, we focused on investigating the impact of IL6 secreted by KCs on the progression and metastasis of HCC.

### IL6 promotes cancer cell migration through the ACAP4-ARF6 pathway

**Figure 3:**
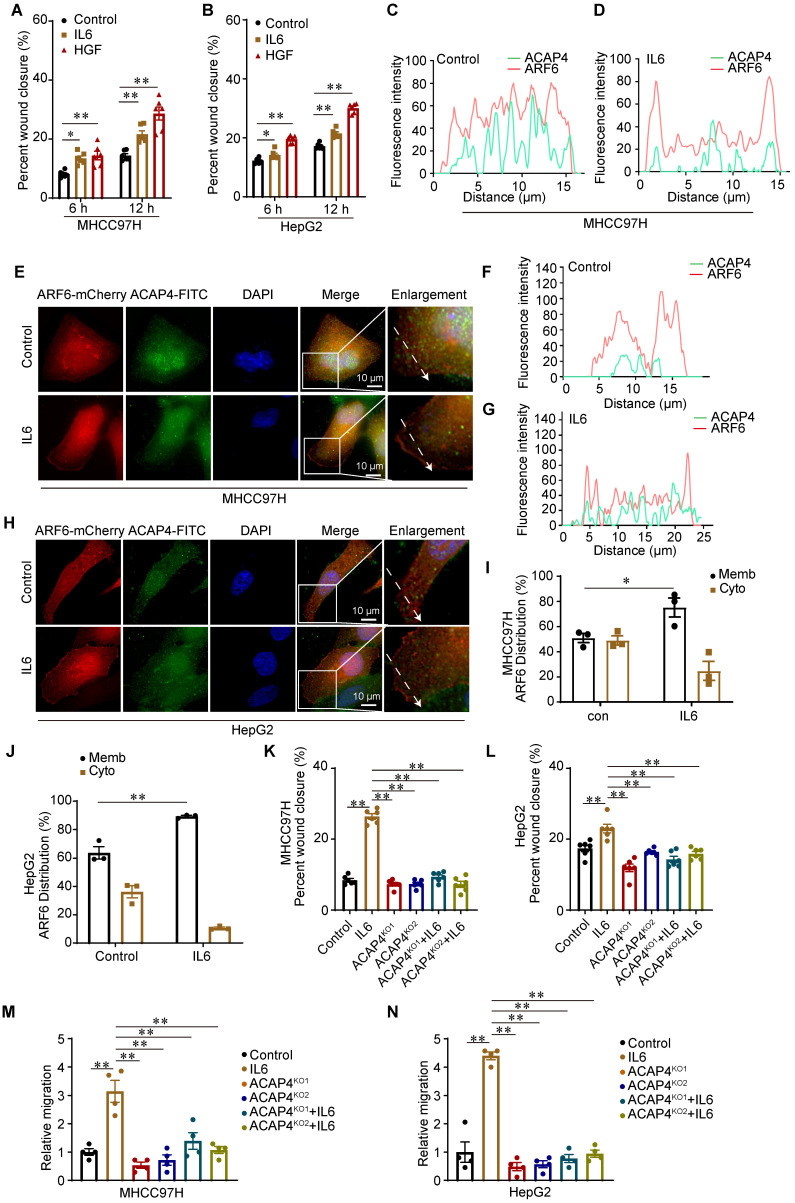
** IL6 promotes cancer cell migration through the ACAP4-ARF6 pathway. (A, B)** Statistical analysis of the migration of MHCC97H and HepG2 cells after IL6 stimulation utilizing wound healing assay. The data are presented as the average ± SEM. *p < 0.05, **p < 0.01. **(C, D)** Quantitative analysis of the fluorescence intensity of ACAP4-FITC and ARF6-mCherry in the control and IL6-treated groups corresponding to the dashed line in the fluorescence images of MHCC97H cells. **(E)** MHCC97H cells were transfected with ARF6-mCherry plasmids for 24 h and then stimulated with IL6 for 20 min. The cells were fixed and stained with an anti-ACAP4 antibody. The images were acquired under a Delta Vision microscope (scale bar, 10 μm). **(F, G)** Quantitative analysis of the fluorescence intensity of ACAP4-FITC and ARF6-mCherry in the control and IL6-treated groups corresponding to the dashed line in the fluorescence images of HepG2 cells. **(H)** HepG2 cells were transfected with ARF6-mCherry plasmids for 24 h and then stimulated with IL6 for 20 min. The cells were fixed and stained with an anti-ACAP4 antibody. The images were acquired under a Delta Vision microscope (scale bar, 10 μm). **(I, J)** Statistical analysis of the ARF6 protein levels in both cytoplasmic and membrane fractions, under conditions with and without IL6 stimulation in MHCC97H and HepG2 cells. **(K, L)** Statistical analysis of the wound healing assay results for MHCC97H and HepG2 cells and ACAP4 KO (ACAP4^KO^) MHCC97H and HepG2 cells stimulated with IL6 (50 ng/mL) for 12 h. The data are presented as the average ± SEM. **p < 0.01. **(M, N)** Statistical analysis of the transwell migration assay results for MHCC97H and HepG2 cells and ACAP4^KO^ MHCC97H and HepG2 cells stimulated with IL6 (50 ng/mL) for 24 h. The data are presented as the average ± SEM. **p < 0.01.

IL6 is a pleiotropic cytokine, and persistent activation of the IL6 signaling pathway is implicated in the occurrence and progression of liver cancer. To examine the function of IL6-induced cell migration, we performed wound healing assay in which MHCC97H cells and HepG2 cells were treated with IL6 (50 ng/mL) or HGF (50 ng/mL) for 12 h. We found that IL6 promoted hepatoma cell migration at 6 and 12 h (*p*<0.01) (Figure 3A-3B; Figure S4A-4B). ACAP4, an ADP-ribosylation factor 6 (ARF6) GTPase-activating protein, was first identified in HCC tissues and was implicated in carcinoma cell movement and invasion through interaction with ARF6 14,26,27. To localize ACAP4 and ARF6 in HCC cells with or without IL6 stimulation, MHCC97H and HepG2 cells were transfected with the ARF6-mCherry plasmid and stained with an anti-ACAP4 antibody. IL6 stimulation triggered the redistribution of ARF6 from the cytoplasm to the plasma membrane in MHCC97H (Figure 3C-3E) and HepG2 cells (Figure 3F-3H). Furthermore, we used a membrane and cytosolic protein extraction kit to isolate membrane and cytosolic proteins from MHCC97H and HepG2 cells, both with and without IL6 stimulation. These findings confirmed that IL6 stimulation facilitates the translocation of ARF6 from the cytoplasm to the plasma membrane (Figure 3I-3J; Figure S4C-4D).

To determine whether the ACAP4 protein is essential for IL6-induced hepatoma cell migration, we knocked out *ACAP4* in MHCC97H and HepG2 cells (ACAP4^KO^) via CRISPR-mediated technology (Figure S4E-4F). To assess the influence of *ACAP4*^KO^ on IL6-induced cell migration, we determined cell migration using wound healing assay and transwell migration assay. Our study demonstrated that IL6 stimulation enhances the migratory capacity of MHCC97H and HepG2 cells. In contrast, ACAP4^KO^ cells exhibited impaired migratory ability, irrespective of IL6 stimulation (Figure 3K-3N; Figure S4G-4I).

### ACAP4 interacts with and is phosphorylated by JAK1 in response to IL6 stimulation

**Figure 4:**
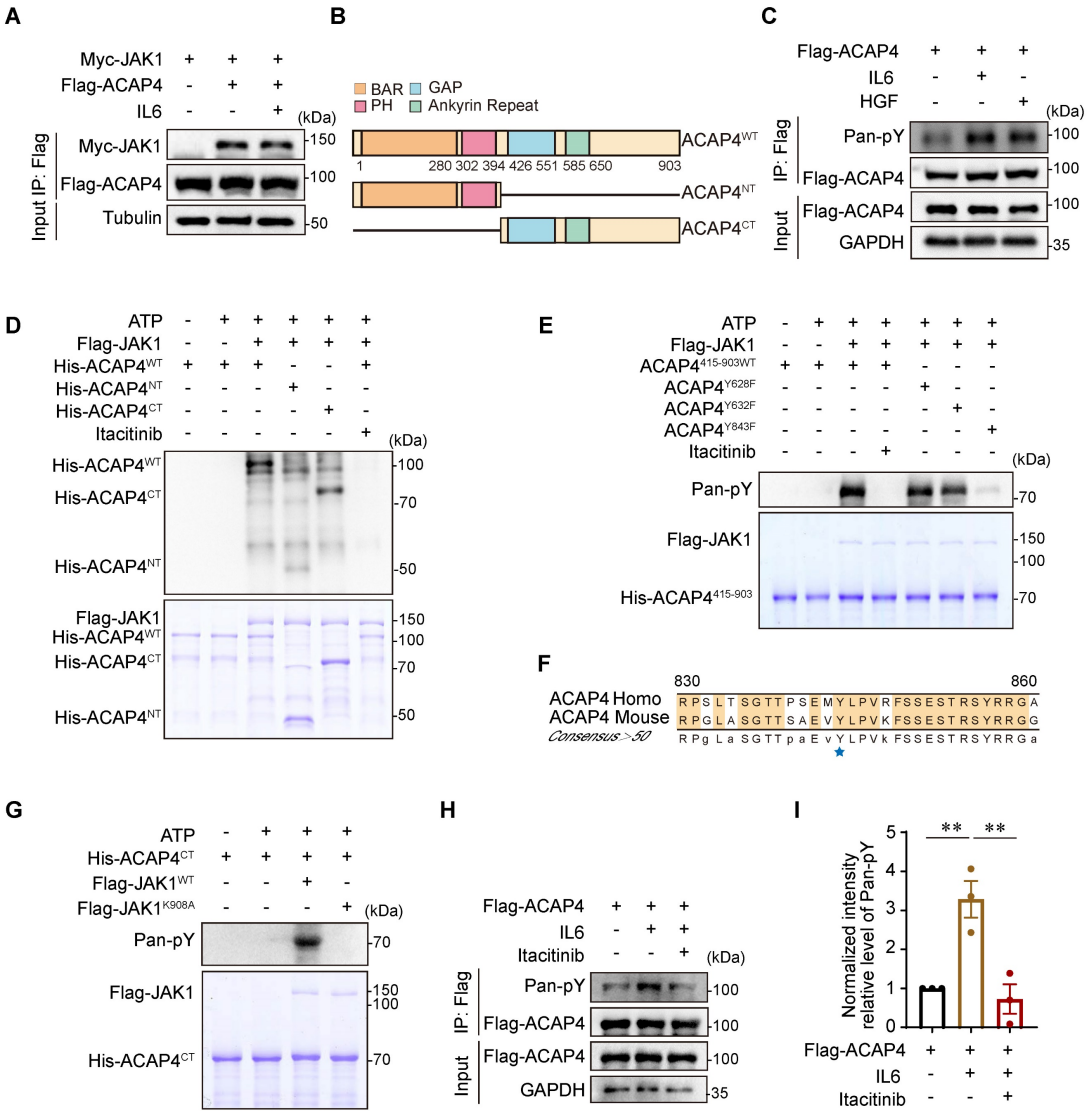
**ACAP4 interacts with and is phosphorylated by JAK1 in response to IL6 stimulation. (A)** MHCC97H cells were co-transfected with Flag-tagged ACAP4 and Myc-tagged JAK1 constructs. Subsequent immunoprecipitation was performed using anti-Flag beads, with or without IL-6 stimulation. The protein levels of Myc-JAK1 were subsequently assessed using an anti-Myc antibody. **(B)** Schematic diagram of ACAP4 protein structural features and truncations. **(C)** MHCC97H cells were transfected with Flag-ACAP4 for immunoprecipitation, and the tyrosine phosphorylation level of ACAP4 was analyzed by IB with an antibody against pan-pY. **(D)** Bacterially expressed His-ACAP4^WT^, His-ACAP4^1-415^ (His-ACAP4^NT^), His-Trx-ACAP4^415-903^ (His-ACAP4^CT^), and 293T cells that expressed Flag-JAK1 were purified and used for the *in vitro* kinase assay. The ACAP4 tyrosine phosphorylation level was analyzed by IB with a pan-anti-pY antibody. **(E)** Bacterially expressed His-Trx-ACAP4^415-903^(ACAP4^415-903WT^), His-Trx-ACAP4^415-903-Y628F^ (ACAP4^Y628F^), His-Trx-ACAP4^415-903-Y632F^ (ACAP4^Y632F^), His-Trx-ACAP4^415-903-Y843F^ (ACAP4^Y843F^), and 293T cells that expressed Flag-JAK1 were purified and used for the *in vitro* kinase assay. The ACAP4 tyrosine phosphorylation level was analyzed by IB with a pan-pY antibody. **(F)** The sequence alignment of ACAP4^830-860^ and Y843 is highlighted with a blue star. (**G)** Bacterially expressed His-Trx-ACAP4^CT^ and 293T cells that expressed Flag-JAK1, as well as Flag-JAK1^K908A,^ were purified and used for the* in vitro* kinase assay. The ACAP4 tyrosine phosphorylation level was analyzed by IB with a pan anti-pY antibody. **(H, I)** MHCC97H cells were transfected with Flag-ACAP4 for IP after itacitinib pretreatment for 2 h and IL6 stimulation for 20 min, and the tyrosine phosphorylation level of ACAP4 was analyzed by IB with an antibody against pan-pY. All experiments were performed on three independent occasions, and the data are presented as the average ± SEM. **p < 0.01.

Aberrant hyperactivation of IL6 signaling and its downstream JAK-STAT3 pathway plays a prominent role in tumorigenesis and metastasis. IL6 signaling involves the engagement of gp130, the dimerization of which leads to the activation of JAKs. The JAK family contains four tyrosine kinases, including JAK1, JAK2, JAK3, and TYK2, of which JAK1 plays a major role in the JAK-STAT signal transduction pathway in response to IL6 28. To determine whether ACAP4 binds to JAK1, MHCC97H cells were cotransfected with Flag-ACAP4 and Myc-JAK1 for coimmunoprecipitation (Co-IP), and the cells were collected 10 min after IL6 (50 ng/mL) stimulation. Myc-JAK1 bound to ACAP4 with or without IL6 stimulation, with no difference between the two groups (Figure 4A, Figure S5A). ACAP4 is a protein of 903 amino acids and contains a Bar domain, a PH domain, a GAP motif, and two ankyrin (ANK) repeats (Figure 4B). On the basis of the characteristics of the ACAP4 structural domain, full-length Flag-ACAP4, and truncations of FLAG-ACAP4, including Flag-ACAP4^1-289^, Flag-ACAP4^290-400^, Flag-ACAP4^401-560^, and Flag-ACAP4^561-903^, were generated and coexpressed with Myc-JAK1 in HEK293T cells (Figure S5B). We conducted Co-IP assays utilizing anti-Flag and anti-Myc antibodies, followed by western blot analysis. Our results demonstrated that Myc-JAK1 associated with Flag-ACAP4^290-400^, Flag-ACAP4^401-560^ and Flag-ACAP4^561-903^ but not with Flag-ACAP4^1-289^ (Figure S5C-5D).

To further investigate the molecular mechanism underlying the function of ACAP4 in IL6-induced cell migration, we performed immunoprecipitation followed by immunoblotting (IP-IB) analyses by using a pan-anti-Tyr phosphorylation (pan-pY) antibody. We found that ACAP4 was phosphorylated in response to IL6 (Figure 4C).

To identify the phosphorylation site of ACAP4 at tyrosine residues, we performed an *in vitro* kinase assay using Flag-JAK1, which was purified from HEK293T cells, and wild-type (WT) His-Trx-ACAP4 (His-ACAP4^WT^), His-Trx-ACAP4^1-415^ (His-ACAP4^NT^), and His-Trx-ACAP4^415-903^ (His-ACAP4^CT^), which were purified from *E. coli* in the absence or presence of the selective JAK1 inhibitor itacitinib 29. IB analyses using the pan-pY antibody confirmed the tyrosine phosphorylation of His-ACAP4^WT^ and His-ACAP4^CT^ but not His-ACAP4^NT^ (Figure 4D).

Our proteomic analyses of His-ACAP4^CT^ identified Tyr628, Tyr632, and Tyr843 as the phosphorylated Tyr residues in ACAP4 (Figure S6A-6C). To further validate the IL6-induced phosphorylation of these Tyr residues in ACAP4 proteins, we performed *in vitro* kinase assays again using purified His-Trx-ACAP4^415-903WT^ (ACAP4^415-903WT^), the nonphosphorylatable mutants His-Trx-ACAP4^415-903-Y628F^ (ACAP4^Y628F^), His-Trx-ACAP4^415-903-Y632F^ (ACAP4^Y632F^) and His-Trx-ACAP4^415-903-Y843F^ (ACAP4^Y843F^) and identified the Tyr843 residue as the major phosphorylation site of ACAP4 induced by IL6 in HCC cells (Figure 4E). Notably, Tyr843 is conserved in humans and mice (Figure 4F). To determine whether IL6 stimulates ACAP4 phosphorylation by activating JAK1, but not JAK2 or TYK2, which share similar protein structures, we performed an *in vitro* kinase assay again using wild-type (WT) Flag-JAK1 (Flag-JAK1^WT^), catalytically inactivated Flag-JAK1 (Flag-JAK1^K908A^), and ACAP4^415-903WT^ (Figure 4G). In addition, we utilized itacitinib to selectively inhibit JAK1, and IP-IB analysis revealed that itacitinib impeded the phosphorylation of Tyr residues in ACAP4 proteins (Figure 4H-4I). IF staining reveals a substantial co-localization of JAK1 and ACAP4 in both the control and IL6-treated groups in MHCC97H and HepG2 cells (Figure S7A-7F). Our results confirmed that JAK1 is the kinase that mediates IL6-induced ACAP4 phosphorylation.

### IL6-induced ACAP4 phosphorylation promotes ARF6-GTPase activity and cancer cell migration

**Figure 5:**
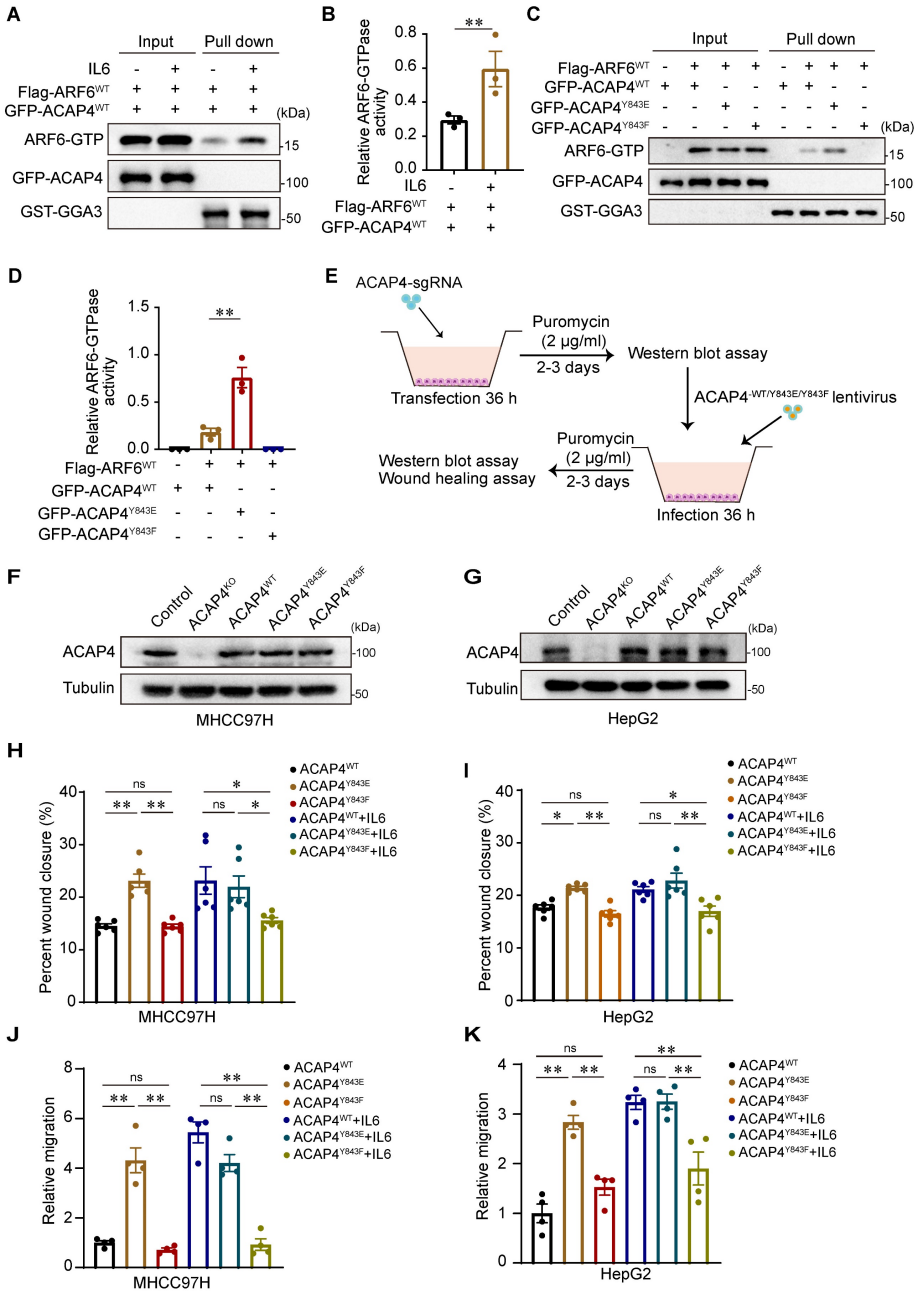
** IL6-induced ACAP4 phosphorylation promotes ARF6-GTPase activity and cancer cell migration. (A)** Endogenous ARF6-GTPase activity was measured by a GGA3 pull-down assay with IL6 simulation. 293T cells coexpressing Flag-ARF6 and GFP-ACAP4^WT^ were incubated with GST-GGA3 after IL6 stimulation. The active forms of ARF6 were measured with an anti-ARF6 antibody. **(B)** Quantitative analyses of ARF6-GTPase activity are described in (**A**). The data are presented as the average ± SEM. **p < 0.01. **(C)** ARF6-GTPase activity was measured via a GGA3 pull-down assay with different ACAP4 mutants. 293T cells that coexpressed Flag-ARF6 and GFP-ACAP4^WT^, GFP-ACAP4^Y843E^, or GFP-ACAP4^Y843F^ were incubated with GST-GGA3. The active forms of ARF6 were measured with an anti-ARF6 antibody. **(D)** Quantitative analyses of ARF6-GTPase activity are described in (C). The data are presented as the average ± SEM. **p < 0.01. **(E)** Flowchart of the construction of the ACAP4^KO^ cell line and stable expression of the exogenous ACAP4^WT^, ACAP4^Y843E^, and ACAP4^Y843F^ cell lines in hepatoma cells. **(F, G)** ACAP4^KO^ MHCC97H and HepG2 cells were infected with lentivirus and expressed ACAP4^WT^, ACAP4^Y843E^, or ACAP4^Y843F^; then, the expression of exogenous ACAP4^WT^, ACAP4^Y843E^, or ACAP4^Y843F^ was assessed by IB. **(H, I)** Quantitative analysis of wound healing in ACAP4^KO^ MHCC97H and HepG2 cells expressing ACAP4^WT^, ACAP4^Y843E^, or ACAP4^Y843F^, with or without IL6 stimulation. **(J, K)** Quantitative analysis of transwell migration assay in ACAP4^KO^ MHCC97H and HepG2 cells expressing ACAP4^WT^, ACAP4^Y843E^, or ACAP4^Y843F^, with or without IL6 stimulation. The data are presented as the average ± SEM. *p < 0.05, **p < 0.01, ^ns^*p* > 0.05.

ACAP4, a novel ARF6 GTPase-activating protein, is essential for ARF6-dependent cell migration through ACAP-ARF6-induced membrane‒cytoskeleton interactions. To determine whether IL6 stimulation modulates the activity of ARF6 GTPase, we used a pull-down assay followed by IB analysis using GGA3^GAT^ as an affinity matrix to capture active ARF-GTP from MHCC97H cells cotransfected with WT Flag-ARF6 (Flag-ARF6^WT^) and WT GFP-ACAP4 (GFP-ACAP4^WT^) after IL6 treatment. We found that IL6 stimulation increased ARF6 GTPase activity (*p*<0.01; Figure 5A-5B). Furthermore, we performed GGA3^GAT^ pull-down assays in MHCC97H cells that were cotransfected with Flag-ARF6 and GFP-ACAP4^WT^, GFP-ACAP4^Y843E^ (a mutant that mimics phosphorylation), or GFP-ACAP4^Y843F^ (a mutant that is nonphosphorylatable) and assessed whether the phosphorylation of ACAP4^Y843^ modulates the activity of ARF6 GTPase. Our data revealed that, compared with GFP-ACAP4^WT^, GFP-ACAP4^Y843E^ significantly upregulated ARF6 GTPase activity (Figure 5C-5D).

To evaluate the impact of ACAP4^Y843^ phosphorylation on cell migration, we re-expressed lentiviral-encoded exogenous ACAP4^WT^, ACAP4^Y843E^, ACAP4^Y843F^ or an empty vector in MHCC97H and HepG2 ACAP4^KO^ cells (Figure 5E-5G). We found that exogenous expression of ACAP4^WT^ or ACAP4^Y843E^ but not ACAP4^Y843F^ rescued ACAP4 KO-impaired IL6-induced cell migration (Figure 5H-5K; Figure S8A-8C). These data indicate that the IL6-induced phosphorylation of ACAP4 at Tyr843-induced hepatoma cell migration is mediated by IL6-JAK1 signaling.

### JAK1-ACAP4^Y843^ is phosphorylated in HCC tissues and may be a biomarker for predicting HCC metastasis

**Figure 6:**
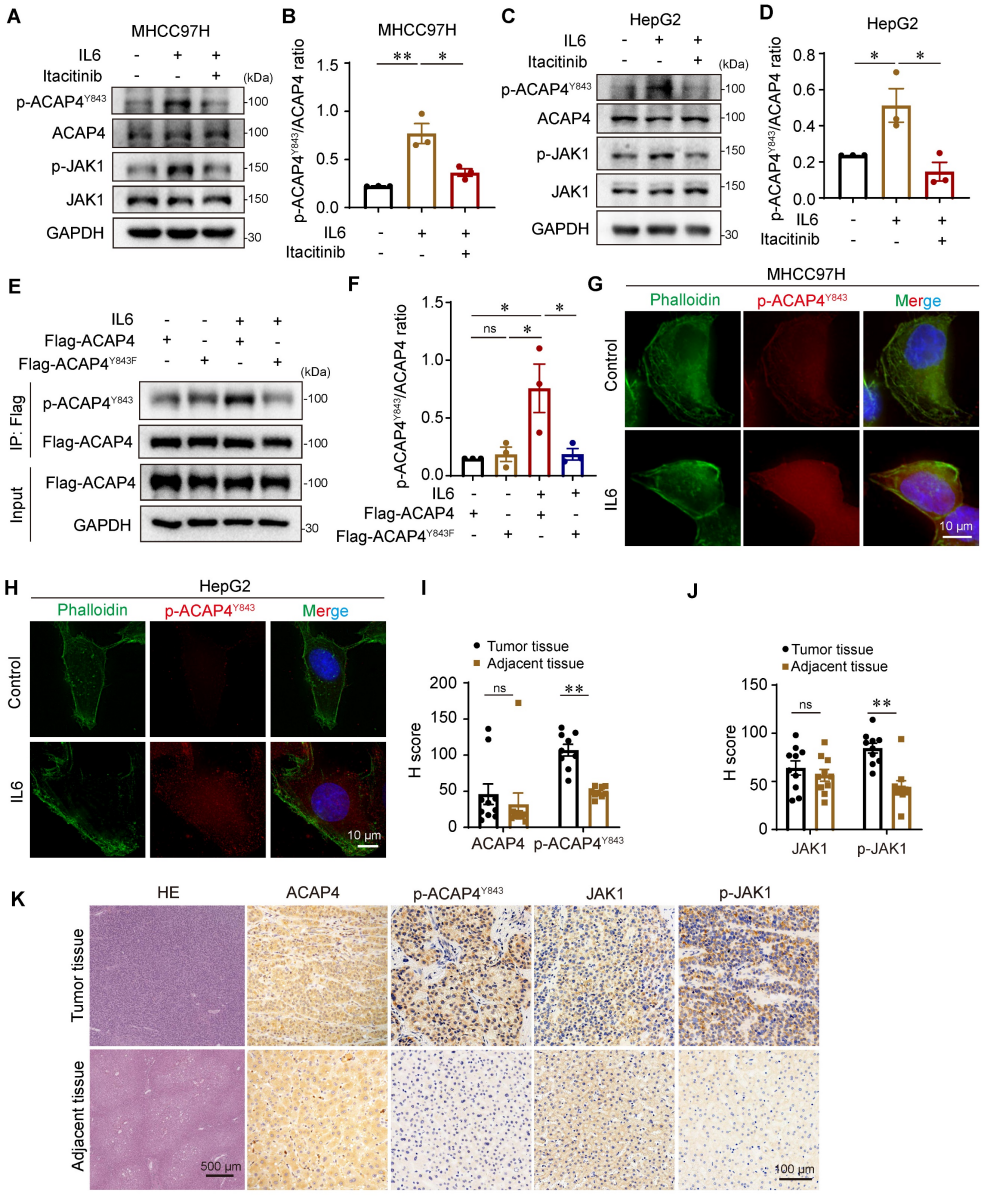
** ACAP4^Y843^ is phosphorylated in HCC tissues and may be a biomarker for predicting HCC metastasis. (A, C)** IB analyses of endogenous ACAP4^Y843^/ACAP4 and p-JAK1/JAK1 ratios in MHCC97H and HepG2 cells after IL6 stimulation. **(B, D)** Quantitative analyses of the ACAP4^Y843^/ACAP4 ratio are described in (**A, C**). The data are presented as the average ± SEM. *p < 0.05, **p < 0.01. **(E)** MHCC97H cells were transfected with Flag-ACAP4 or Flag-ACAP4^Y843F^ for IP after IL6 stimulation, and the tyrosine phosphorylation level of ACAP4^Y843^ was analyzed by IB with an anti-p-ACAP4^Y843^ antibody. **(F)** Quantitative analyses of the ACAP4^Y843^/ACAP4 ratio described in (**E**). The data are presented as the average ± SEM. *p < 0.05, ^ns^*p* > 0.05. **(G, H)** MHCC97H and HepG2 cells were starved of serum for 4 h before being stimulated with IL6 for 20 min. Then, the cells were fixed, permeabilized, and stained with the p-ACAP4^Y843^ antibody. The images were acquired under a Delta Vision microscope (scale bar, 10 μm). **(I, J)** Statistical analysis of the expression levels of ACAP4, p-ACAP4^Y843^, JAK1 and p-JAK1 are described in (**K**). The data are presented as the average ± SEM. **p < 0.01, ^ns^*p* > 0.05. **(K)** HE staining and expression levels of ACAP4, p-ACAP4^Y843^, JAK1, and p-JAK1 in tumor tissues and adjacent tissues from representative patients with HCC were detected by immunohistochemistry (scale bar, 100 μm).

The phosphorylation of ACAP4 at Tyr843 is increased in HCC with hyperactivation of IL6-JAK1 signaling, which could be an important marker for predicting the metastasis of HCC. To this end, we generated a rabbit polyclonal anti-p-ACAP4^Y843^ antibody and tested the endogenous protein level of ACAP4 phosphorylated at Tyr843 in the absence or presence of IL6 stimulation in hepatoma cells. The results revealed high expression of p-ACAP4^Y843^ after IL6 stimulation compared with that in the control group. JAK1 inhibition by itacitinib abolished the phosphorylation of ACAP4^Y843^ in MHCC97H (Figure 6A-6B) and HepG2 cells (Figure 6C-6D).

Next, we performed IP-IB analyses of HEK293T cells expressing Flag-ACAP4^WT^ and Flag-ACAP4^Y843F^. We found that IL6 treatment induced phosphorylation at the Tyr843 residue in WT ACAP4 proteins. Moreover, weak phosphorylation of the ACAP4 protein in Flag-ACAP4^Y843F^-expressing HEK293T cells could be due to the increased phosphorylation of p-Y843 in the endogenous WT ACAP4 protein (Figure 6E-6F). In addition, we tested the intracellular localization of p-ACAP4^Y843^ in the absence or presence of IL6 stimulation. IF with an antibody against p-ACAP4^Y843^ revealed the distribution of p-ACAP4^Y843^ in the cytoplasm and plasma membrane in MHCC97H and HepG2 cells, which coincided with the localization of ACAP4. IL6 stimulation increased the protein level of p-ACAP4^Y843^ in the cytoplasm and plasma membrane (Figure 6G-6H).

To further evaluate the clinical value of p-ACAP4^Y843^ in HCC, we performed HE and immunohistochemical staining of HCC tumor tissues and adjacent tissues using antibodies against ACAP4, p-ACAP4^Y843^, JAK1, and p-JAK1 (Figure 6I-6K). As expected, p-ACAP4^Y843^ and p-JAK1 were highly expressed in HCC tumor tissues compared with adjacent tissues. However, there were no significant differences in ACAP4 or JAK1 protein levels between tumor tissues and adjacent tissues.

### Bufalin, an active ingredient from Venenum Bufonis used to treat HCC, targets JAK1 to inhibit ACAP4^Y843^ phosphorylation

JAK1 plays a central role in ACAP4^Y843^-regulated cell migration in response to IL6. Moreover, activation mutations in JAK1 and STAT3 frequently occur in HCC 30,31. Selective JAK1 inhibition can be a useful approach for blocking oncogenic IL6-JAK1 signaling. Natural products and traditional Chinese medicine (TCM) have long been used for HCC prevention and treatment in East Asian countries. Accumulating clinical evidence and fundamental research have revealed these effectiveness in limiting HCC progression and preventing postoperative recurrence 32,33. To identify the active ingredients for JAK1 inhibition in liver cancer, we analyzed the literature concerning the use of natural products and TCM for the treatment of liver cancer and searched for the main active components using data mining and network pharmacology (Figure S9A). The CCK8 assay was subsequently used to detect the dose range of cytotoxicity of the identified active components on MHCC97H cells (Tables S2A-2B). HCC cells were treated with IL6 (50 ng/mL) for 20 min after pretreatment with a nontoxic dose of the active ingredients for 3 h. Western blotting analyses indicated that bufalin may be an effective ingredient for inhibiting JAK1 activity (Figure S9B-9E).

**Figure 7:**
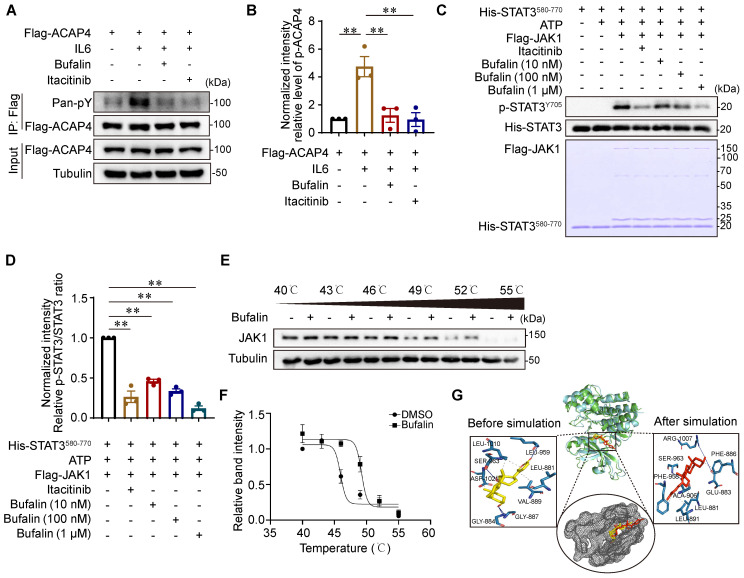
** Bufalin, an active ingredient from Venenum Bufonis used to treat HCC, targets JAK1 to inhibit ACAP4^Y843^ phosphorylation. (A)** MHCC97H cells were transfected with Flag-ACAP4 for IP after bufalin or itacitinib pretreatment and IL6 stimulation for 20 min. The tyrosine phosphorylation level of ACAP4 was analyzed via IB with a pan anti-pY antibody. **(B)** Quantitative analyses of the pY-ACAP4/ACAP4 ratio are described in (**A**). The data are presented as the average ± SEM. **p < 0.01. **(C)** Bacterially expressed His-STAT3^580-770^ and 293T cells expressing Flag-JAK1 were purified and used for the *in vitro* kinase assay after itacitinib or different concentrations of bufalin treatment. The level of p-STAT3^Y705^ phosphorylation was analyzed by IB with an anti-p-STAT3^Y705^ antibody. **(D)** Quantitative analyses of the phosphorylation level of p-STAT3^Y705^ are described in (**C**). The data are presented as the average ± SEM. **p < 0.01. **(E)** Cellular thermal shift assay of bufalin with JAK1. **(F)** Quantitative analyses of the thermal shift assay of bufalin with JAK1. **(G)** Molecular dynamics simulation of the interaction of bufalin with JAK1 according to the structures of bufalin and JAK1.

To determine the inhibitory effect of bufalin on JAK1-ACAP4, we performed IP-IB analyses of MHCC97H cells that were transfected with Flag-ACAP4 and treated with or without bufalin (10 nM) or itacitinib (100 nM). We found that bufalin effectively inhibited the IL6-induced phosphorylation of ACAP4 (*p*<0.01) to an extent similar to that of itacitinib (Figure 7A-7B). To further determine the direct inhibitory effect of bufalin on JAK1 *in vitro*, we performed the *in vitro* kinase assays described above and found that bufalin significantly inhibited JAK1 at concentrations ranging from 10 nM to 1 μM (Figure 7C-7D).

Next, we determined the biophysical interactions between drugs and protein targets. A cellular thermal shift assay (CETSA) was used to evaluate the interaction between bufalin and JAK1 in cells. We obtained the melting curves of JAK1 as well as its CETSA profiles upon bufalin addition. Comparative analysis revealed significant shifts in the fit curve for the JAK1 proteins upon the addition of bufalin (Figure 7E-7F), suggesting that bufalin directly binds to JAK1 in a cellular context.

To gain further insight into the types of interactions between bufalin and the target pocket, we performed molecular docking and dynamics simulations. Through a detailed examination of the interaction profile of the resulting docked complex, we identified a number of hydrophobic interactions as well as specific hydrogen bond formations (Figure S10A-10B). We focused more on the hydrogen bond interactions, as they are generally responsible for the higher structures of proteins and their interactions with ligands. The RMSD trajectories were analyzed to reveal the structural changes that occur in protein structures over a specified time frame in comparison to the starting structure. The lower degree of deviation of the trajectory representing the protein‒ligand complex compared with the apo structure is an indicator of complex stability. The flattening and levelling off of the RMSD complex are also indicators of protein equilibration, indicating the stability of bufalin upon complex formation with JAK1 (Figures S10C-10D). Additionally, the RMSF represents deviation at specific positions, while the radius of gyration symbolizes the degree of compactness of macromolecular structures, and the SASA is the geometric estimation of the degree of exposure of surface atoms of proteins to solvents. The resulting outputs from these analyses, which were consistent with the calculated RMSD of both systems, collectively suggest complex stability (Figure S10E-10G).

Using the initial postdocking single conformation structure of the bufalin-JAK1 complex, four active site residues (Gly884, Gly887, Leu959, and Ser963) were observed to be involved in the interaction of the hydrogen bond with bufalin. Next, the system was subjected to dynamic simulation, and the interaction profile throughout this period was analyzed. We then extracted the pose of the last frame from the simulation trajectory for another round of interaction profile analysis. From this frame, Ser963 along with Phe886 and Arg1007 were the only residues shown to form hydrogen bond interactions with bufalin (Figure 7G), suggesting that this set of active site residues may be essential for the stable binding of bufalin.

### Bufalin inhibits cell migration and tumor metastasis in HCC cells and orthotopic xenograft mice

**Figure 8:**
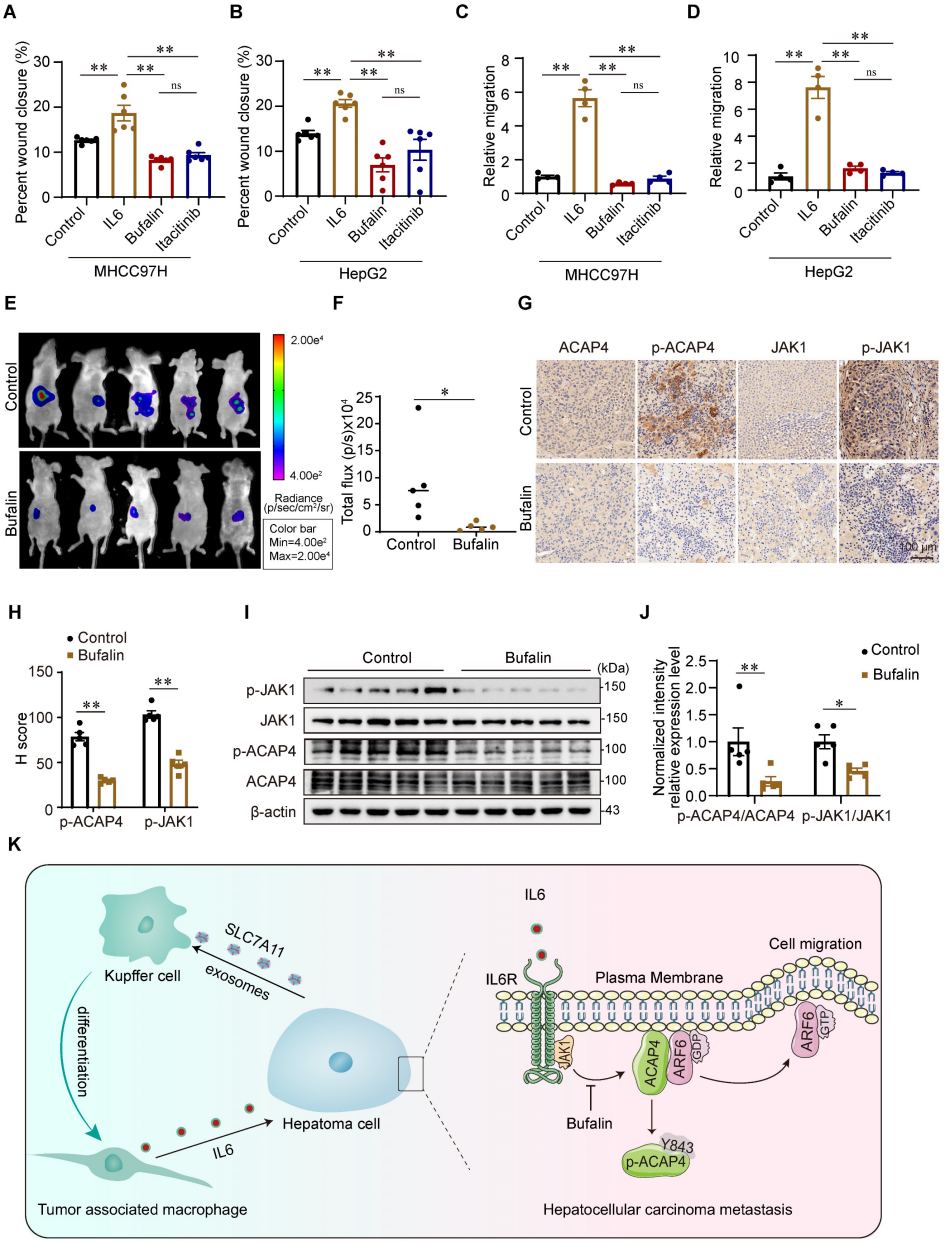
** Bufalin inhibits cell migration and tumor metastasis in HCC cells and orthotopic xenograft mice. (A, B)** Quantitative analyses were conducted on the wound healing assay involving MHCC97H and HepG2 cells that were pretreated with bufalin or itacitinib, followed by stimulation with IL6 at a concentration of 50 ng/mL for a duration of 12 h. The data are presented as the average ± SEM. **p < 0.01, ^ns^*p* > 0.05. **(C, D)** Quantitative analyses were conducted on the transwell migration assay involving MHCC97H and HepG2 cells that were pretreated with bufalin or itacitinib, followed by stimulation with IL6 at a concentration of 50 ng/mL. The data are presented as the average ± SEM. **p < 0.01, ^ns^*p* > 0.05. **(E)** Bioluminescence images of MHCC97H-luciferase cell orthotopic xenograft mice. **(F)** Quantification of bioluminescent signals (photons/sec) from HCC orthotopic xenograft mice. The data are presented as the average ± SEM. n=5. *p < 0.05. **(G)** The expression levels of ACAP4, p-ACAP4^Y843^, JAK1, and p-JAK1 in tumor tissues from representative HCC orthotopic xenograft mice were detected by immunohistochemistry (scale bar, 100 μm). **(H)** Statistical analysis of the expression levels of p-ACAP4^Y843^ and p-JAK1 in tumor tissues from orthotopic xenograft mice. The data are presented as the average ± SEM. **p < 0.01. **(I)** The expression levels of ACAP4, p-ACAP4^Y843^, JAK1, and p-JAK1 in tumor tissues from representative HCC orthotopic xenograft mice were detected by IB. **(J)** Statistical analysis of the expression levels of p-ACAP4^Y843^ and p-JAK1 in tumor tissues from orthotopic xenograft mice. The data are presented as the average ± SEM. *p < 0.05, **p < 0.01. **(K)** The model illustrates the proposed role of IL6 from HCC-educated KCs in regulating HCC metastasis via the JAK1‒ACAP4 pathway.

To further evaluate whether bufalin inhibits HCC tumor metastasis *in vitro* and *in vivo* by targeting JAK1-ACAP4, we performed wound healing and transwell migration assays to assess the inhibitory effect of bufalin on IL6-induced hepatoma cell migration. Compared with IL6 stimulation alone, bufalin (10 nM) significantly inhibited IL6-stimulated MHCC97H and HepG2 cell migration (Figure 8A-8D; Figure S10H-10J). Then, we constructed HCC orthotopic xenograft mice. The results revealed that intraperitoneal injection of bufalin for 2 weeks substantially suppressed tumor metastasis in nude mice bearing HCC tumor xenografts (Figure 8E-8F). Bufalin also inhibited the phosphorylation of JAK1 and ACAP4^Y843^ (Figure 8G-8J).

In conclusion, this study describes a new mechanism by which IL6 secreted from KCs promotes HCC metastasis. Liver KCs are polarized to TAMs by the induction of hepatoma cell-derived exosomes, which then secrete IL6 and activate JAK1 in hepatoma cells. JAK1 activation phosphorylates ACAP4 at Tyr843, thereby increasing ARF6-GTPase activity to promote hepatoma cell migration and HCC metastasis. Bufalin, an active ingredient from natural products used to treat HCC, targets JAK1-ACAP4 to inhibit hepatoma cell migration and HCC metastasis (Figure 8K).

## Discussion

TAMs are one of the main tumor-infiltrating immune cell types that constitute a plastic and heterogeneous cell population of the TME, accounting for up to 50% of several solid tumors, and are correlated with poor prognosis 34. On the one hand, exosomes derived from tumor cells induce macrophages to differentiate into M2-like TAMs and contribute to the protumorigenic microenvironment through angiogenic and lymphangiogenic regulation and immune suppression. On the other hand, TAM-derived cytokine feedback to tumors facilitates tumor cell migration, invasion, and proliferation. Our data revealed a new mechanism by which liver KC-derived TAMs secrete IL6 to promote HCC metastasis in a JAK1-ACAP4-ARF6 pathway-dependent manner.

KCs constitute the largest population of inherent macrophages in human tissues and constitute an important source of TAMs in HCC 35,36. TAMs accelerate tumor progression and metastasis by releasing inflammatory cytokines, chemokines, and angiogenic factors. Among them, IL-6 is a key inflammatory factor secreted by TAMs in the microenvironment of HCC 20. The IL6 signaling pathway is aberrantly hyperactivated in a variety of malignancies, and a high serum level of IL6 is correlated with increased risk of HCC 37. IL6 drives the compensatory proliferation of hepatocytes, which accumulate DNA damage 21. IL6 also promotes the epithelial-to-mesenchymal transition and activates the STAT3 transcription factor-driven oncogenic gene program 38. In addition, chromosomally unstable cancer cells increase the expression of IL6 and trigger IL6 signaling downstream through the cGAS-STING pathway, thereby promoting cancer cell survival and growth 39. Our results revealed the upregulation of IL6 in both the serum and cancer tissues of HCC patients, which was positively correlated with the protein level of MMP9 and linked to a worse prognosis. scRNA-seq analysis, induction of mouse KCs *in vitro,* and coculture of KCs with exosomes revealed that KCs serve as a significant source of IL6 production within the HCC TME following their differentiation into TAMs induced by exosomes derived from hepatoma cells.

ACAP4, encoded by the *DDEFL1* (development and differentiation enhancing factor-like 1) gene, is a specific GAP of ARF6 involved in membrane cytoskeletal remodeling and cell migration via an ARF6-dependent pathway. ACAP4 interaction with ezrin is also essential for histamine-stimulated apical membrane remodeling and parietal cell secretion 40-43. Our recent studies revealed that the phosphorylation of ACAP4 orchestrates EGF-stimulated integrin β1 recycling during cell migration 15,16. In addition, the acetylation of ACAP4 at Lys311 ensures the dynamic cycling of the ARF6-ACAP4 complex with the plasma membrane and is essential for CCL18-induced breast cancer cell migration and invasion 17. In this study, we show that IL6 elicits cell migration through the JAK1-ACAP4-ARF6 pathway and that JAK1 is the key enzyme in the JAK family that phosphorylates the Tyr843 site of ACAP4 in response to IL6. The phosphorylation of ACAP4^Y843^ promoted ARF6-GTPase activity and hepatoma cell migration, which was consistent with the findings in HCC tissues.

The hyperactivation of JAK1 kinase has been closely associated with a wide range of human cancers, including liver, lung, breast, and gastric cancers 31,44-46. Earlier whole-genome sequencing revealed 7 distinct protein-altering mutations in JAK1 in HCC (accounting for 9.1%), of which five constitutively active mutants increased JAK1 kinase activity compared with WT JAK1 30. As the major enzyme in the JAK family, activated JAK1 subsequently phosphorylates tyrosine residues in the cytoplasmic region of gp130, thereby initiating JAK1/STAT signaling 47. Our results reveal a previously unexplored tumor-promoting mechanism of JAK1 signaling. JAK1 promotes hepatoma cell migration in an ACAP4-ARF6-dependent manner in response to IL6. Notably, JAK1 inhibitors, including tofacitinib, ruxolitinib, and pacritinib, have been broadly investigated in conditions involving chronic inflammation and hematological malignancies, with fewer evaluations in patients with solid tumors 47. Due to the high number of adverse events, studies of the efficacy of JAK1 inhibitors against HCC are still in the preclinical stage 50-52. In East Asia, natural products and TCM are the most widely used form of complementary and alternative medicine, which benefits HCC patients by inhibiting progression, reducing adverse reactions after TACE therapy, and prolonging survival time 53-55. Our search for active components that inhibit JAK1 identified bufalin, an active ingredient from Venenum Bufonis, as having highly inhibitory effects on JAK1 kinase activity. Bufalin directly binds to JAK1 and suppresses hepatoma cell migration and tumor metastasis in HCC orthotopic xenograft mice. Importantly, bufalin may be a promising candidate for the treatment of HCC via the inhibition of the JAK1-ACAP4 pathway.

In summary, we demonstrated that hepatoma cell-derived exosomes induced KCs to differentiate into TAMs, thereby secreting IL6. IL6 was upregulated in both the serum and tumor tissues of patients with HCC and is negatively correlated with poor prognosis. During IL6-induced JAK1 signaling in tumor cells, we identified a novel phosphorylation site of ACAP4 at Tyr843 that facilitates hepatoma cell migration by increasing ARF6-GTPase activity. Additionally, we verified the high levels of p-ACAP4^Y843^ and p-JAK1 in HCC tissues, which may be potential biomarkers indicating HCC metastasis in IL6-highly expressed cell populations in the HCC TME. Moreover, we found that bufalin, an active ingredient from Venenum Bufonis against HCC, targeted JAK1 to impede hepatoma cell migration and HCC metastasis.

## Materials and Methods

### Tissue and serum

Primary HCC tissue and serum samples were obtained from patients who underwent curative resection at the Fifth Medical Center of PLA General Hospital (Beijing, China). All included patients were identified as having HCC by histopathology. Moreover, all patients were devoid of neoadjuvant chemotherapy or radiotherapy before surgical resection and were not diagnosed with cholangiocarcinoma or mixed hepatocellular and cholangiocarcinoma. Peripheral blood samples with a volume of 1 mL from 10 normal elderly persons were collected in EDTA-containing tubes at the Dongzhi Meng Hospital of the Beijing University of Chinese Medicine. All samples were collected with informed consent from patients, and all related procedures were performed with the approval of the internal review and ethics boards of the Beijing University of Chinese Medicine (Approval Number: 2018BZHYLL0103).

### Olink multiplex cytokines assay

Relative immune and oncology-related protein expression in peripheral blood plasma was measured by Olink proximity extension technology. Blood was centrifuged for 15 min at 2000 × g, and serum was collected and aliquoted for storage at -80 °C until analysis. 92 immune and oncology-related proteins were analyzed in the serum using the Olink target immuno-oncology panel. The output was normalized and presented in a relatively semi-quantitative Normalized Protein eXpression unit (NPX) according to the manufacturer's protocols. The serum inflammatory cytokines and chemokines were extracted to analyze the possible biomarkers indicating HCC progression and metastasis.

### Landscape and Dynamics of Single Cells in Hepatocellular Carcinoma

The scRNA-seq data of 46 patients was downloaded from the GEO database (GSE151530). We used the harmony algorithm to integrate scRNA-seq datasets for batch effect correction and unsupervised clustering. We then selected the top 2000 largest variable genes as highly variable genes (HVGs) and performed subsequent analyses such as PCA clustering. “RunPCA” function was used to perform the principal component analysis (PCA). These analyses identified 30 significant principal components. We annotated cell clusters based on the expression of curated known cell markers on the UMAP plot. The statistical method to identify differentially expressed genes was based on the Wilcox rank sum test.

To conduct a correlation analysis between IL6 and overall survival in HCC patients, as well as with TAMs markers, we utilized the TCGA dataset comprising data from 346 HCC patients. Of these 346 patients, 117 had high expression of IL6 and 229 had low expression of IL6. The Pearson correlation coefficient was obtained from the above sources. Potential correlation of IL6 with MMP9 and Arg1 in TCGA datasets was then determined. A p-value lower than 0.05 was considered indicative of a significant survival association.

### Cell culture

MHCC97H, HepG2, THLE-2, and 293T cells were obtained from the American Type Culture Collection (ATCC, USA). MHCC97H, HepG2 and 293T cells were cultured in DMEM (Thermal Fisher, USA) containing 10% FBS (Hyclone, USA), 100 U/mL penicillin (Thermal Fisher), and 100 μg/mL streptomycin (Thermal Fisher). THLE-2 cells were cultured in 90% BEBM^TM^ Bronchial Epithelial Growth Medium (CC-3171, CC-4175, Lonza, USA), 10% FBS, 100 U/mL penicillin (Thermal Fisher), and 100 μg/mL streptomycin (Thermal Fisher).

### Mouse KCs isolation, induction and phagocytic activity assay

Mouse liver KCs were isolated from C57BL/6 mice (Male, 18 ± 2 g) using percoll density gradient centrifugation. After mouse anesthesia, warm HBSS-EGTA was used for perfusion through the inferior vena cava. When the liver swells and becomes discolored, instead of HBSS-EGTA with collagenase buffer, continue perfusing the liver until it is sufficiently digested. Collect the liver and transfer it to a Petri dish containing cold HBSS-CaCl_2_ buffer. Release the hepatic cells by cutting the liver lobes and filtering the cells through a 100 μm cell strainer. Then, centrifuge for 3 min at a speed of 50 ×g at 4 °C. Collect the supernatant containing the KCs for the percoll gradient centrifugation following reference 56. To create the percoll gradient, 15 mL of 25% percoll solution is carefully layered at the bottom of a 50 mL centrifuge tube. Next, 10 ml of 45% percoll solution is slowly introduced by touching the pipette tip to the tube's base, ensuring it is fully seated beneath the 25% percoll layer. The cell-containing supernatant obtained from the centrifugation step is gently layered on top of the percoll gradient using a 1 mL pipette, repeatedly and slowly along the tube walls. The tube is then centrifuged at 4 °C and 1200×g for 30 min without brakes. The intermediate fraction, corresponding to the leukocyte layer, is meticulously extracted using a pipette and transferred to a new 50 mL centrifuge tube. The cells undergo two washing steps, followed by centrifugation at 500×g for 5 min per wash. Subsequently, the KCs pellet is resuspended in 10 mL of RPMI 1640 medium and subjected to another centrifugation at 500 ×g for 5 min.

LPS (50 ng/mL, L2630, Sigma-Aldrich) and IFN-γ (20 ng/mL, C746, Novoprotein) were used to induce M1-type polarization after 24 h of treatment. IL4 (10 ng/mL, 214-14, PeproTech) and IL13 (10 ng/mL, 210-13, PeproTech) were used to induce M2-type polarization after 48 h of treatment.

To assess the phagocytic activity of KCs, isolated cells were plated in 24-well plates and allowed to attach for 1 to 2 days. Subsequently, ink was diluted to a ratio of 1:800 using PBS and introduced to the cell culture wells. The cells' phagocytic response was monitored and captured using an inverted optical microscope.

### Exosomes isolation and proteomic analysis

The culture medium of MHCC97H and THLE-2 cells was replaced with a medium containing 10% exosome-depleted FBS (50A-1, SBI, USA) before the exosomes were isolated from the cell supernatant. After 48 h of incubation, the conditioned medium was collected in a 50 mL Falcon tube using a sterile pipet and centrifuged at 300 × g for 10 min to remove the cell pellets. Then, transfered the cleared conditioned medium to a new 50 mL Falcon tube and centrifuge for 20 min at 2000 × g at 4 °C; transfered the supernatant to new polyallomer tubes for ultracentrifugation for 30 min at 10000 × g at 4 °C (L-100XP, Beckman, USA); transfered the supernatant to a new tube and centrifuge the supernatant for 70 min at 100000 × g, at 4 °C. The pellets were resuspended in PBS and subjected to centrifugation for 70 min at 100000 × g at 4 °C. The supernatant was removed and the pellet was resuspend in 200-500 μL PBS. The resulted exosomes were stored at -80 °C as 50 μL aliquots for the following analysis. The proteomic analysis of exosomes derived from MHCC97H and THLE-2 cells was conducted by Shanghai Wayen Biotechnologies, Inc (China).

### RNA sequencing and quantitative real-time PCR (qRT-PCR)

The cells were washed with PBS three times and lysed with RNA lysis buffer (Promega). Extracted RNA was mixed with purification beads to obtain RNA with higher purity. After passed the quality inspection, the following library construction and sequencing were completed by Shanghai OE Biotech Co., Ltd. (China). Mouse KCs treated with MHCC97H or THLE-2 cell-derived exosomes for 48 h were lysed with RNA lysis buffer (Promega). The RT-PCR reaction was prepared by AceQ qPCR SYBR Green Master Mix (Q111, Vazyme) following the manufacturer's instructions. The delta CT value was calculated based on the CQ value of the target gene and the GAPDH gene. The difference in gene expression was expressed as a fold change.

### Plasmid construction, sgRNA, Lentivirus construction and infection

ACAP4-GFP and ARF6-mCherry were constructed as described previously 16. The JAK1-Myc plasmid was purchased from Miaolingbio (China). pCMV-24-3×Flag (OGS622) was from Sigma. Constructs of Flag- and His-tagged proteins and truncations were generated by amplifying the indicated fragment of the gene by PCR and then inserted into pCMV-24-3×Flag (Sigma) and pET-22b (Novagen), respectively. Site-directed mutants of Flag- and His-tagged ACAP4 and JAK1 were generated by the Mut Express II Fast Mutagenesis Kit (Vazyme) according to the manufacturer's instructions. The sequence for the N-terminal GGA3 (1-303) was amplified by PCR and inserted into the pGEX-6P-1 vector to generate the GST-fusion protein GGA3^GAT^.

For endogenous ACAP4 gene knockout experiments, the sgRNA primers were designed through https://cctop.cos.uni-heidelberg.de:8043/. The following sgRNA sequences target ACAP4 gene knockout:

ACAP4-sg1-F: CACCGCATCCTGCAGAGAATAAAGA;

ACAP4-sg1-R: AAACTCTTTATTCTCTGCAGGATGC;

ACAP4-sg2-F: CACCGCTAAGGATTCCACGGCCTCT;

ACAP4-sg2-R: AAACAGAGGCCGTGGAATCCTTAGC.

For stable expression of exogenous ACAP4^WT^, ACAP4^Y843F^, and ACAP4^Y843E^, the indicated fragment of the gene was amplified by PCR and then inserted into the lentivirus-based pLVX vector (Addgene, USA). Hepatoma cells were transfected with sgRNA for 36 h, then 2 μg/mL puromycin was added to screen out the cells. The lentivirus-based vectors pLVX, psPAX2, and pMD2.G were co-transfected into the HEK293T cells by lipofectamine 3000 (L3000150, Thermal Fisher) for 48 h, and the supernatant medium was harvested. For MHCC97H cell infection, the medium was supplemented with lentivirus at a ratio of 1:1 and supplemented with 8 µg/mL polybrene (Sigma), and incubated for 12 h. Then, the medium was replaced with the normal medium. After 36 h, 2 μg/mL puromycin was added to filter out the cells that were resistant to puromycin.

### Recombinant protein expression and purification

His-tagged ACAP4 wild-type, mutants, truncations, His-tagged STAT3^580-770^, and GST-tagged GGA3^GAT^ were expressed in *E. coli* strain Rosetta (DE3). 0.5 mM isopropyl β-d-1-thiogalactopyranoside was used to induce protein expression overnight at 16 °C. Bacteria pellets expressed His-tagged proteins were lysed in cold lysis buffer (50 mM NaH_2_PO4, pH 8.0; 300 mM NaCl, 10 mM imidazole, 5% glycerol, 1 mM PMSF), and then the solution was bound with Ni-NTA agarose (Qiagen, USA) and eluted with elution buffer (50 mM NaH_2_PO4, pH 8.0; 300 mM NaCl, 200 mM imidazole). Bacteria pellets expressing GST-tagged proteins were lysed in cold PBS containing 0.5% TritonX-100 and protease inhibitor cocktail (Sigma) and incubated with Glutathione Sepharose 4B (Sigma). The GST-tagged protein was eluted with PBS containing 20 mM reduced glutathione and identified by SDS-PAGE.

### Western blot, Immunoprecipitation and GGA3 pull-down assay

The protein samples were separated by SDS-PAGE and then transferred onto nitrocellulose membranes. The membranes were sequentially blocked in TBST containing 5% skim milk, incubated with the primary antibody, and then incubated with the second antibody. The bands in the membrane were visualized using the chemiluminescence reagent (ThermoFisher) and quantified by the Imaging System (Qingxiang, China). HEK 293T cells transfected with the indicated plasmids were lysed in lysis buffer. Flag M2 beads (Sigma) or anti-Myc Agarose Gel (Beyotime) were used to incubate the lysates at 4 °C for 3 h. After being washed with lysis buffer three times, the beads were boiled, and the bound proteins were tested by western blot. The ARF6-GTPase activity was measured by the GST-GGA3 pull-down assay as previously described 57. The ARF6-GTPase activity was analyzed by comparing the protein level of precipitated ARF6 using an ARF6 antibody.

### Separation of cytoplasmic and membrane proteins

To isolate cytoplasmic and membrane proteins, cultured cells were scraped, centrifuged at 600 ×g for 5 min at 4 °C, and the pellet was resuspended in the PMSF-containing extraction reagent (Beyotime, P0033). After mixing and incubating on ice for 10 min, the suspension underwent four freeze-thaw cycles between liquid nitrogen and room temperature to break the cells. The sample was centrifuged at 700 ×g for 10 min to collect the supernatant. It was then centrifuged at 4 °C, 14000 ×g for 30 min to obtain the cytoplasmic protein fraction. Membrane protein reagent B was added to the pellet, incubated on ice, and centrifuged at 14000 ×g for 5 min to collect the membrane protein fraction. Proteins were analyzed by western blot.

### *In vitro* kinase assay and Mass spectrometry

Flag-tagged JAK1 wild-type and K908A mutants were purified from HEK293T cells using Flag M2 beads. His-tagged ACAP4 wild-type, mutants, truncations, and His-tagged STAT3^580-770^ were purified as described above. Purified JAK1-Flag and indicated proteins were incubated in kinase buffer×2 (50 mM Tris-HCl, pH 7.5; 10 mM MgCl_2_, 1 mM EGTA, 1 mM DTT, 500 μM ATP, 1 μM NaF) at 37 °C for 45 min. The SDS-PAGE sample buffer was added to stop the reaction, and the samples were boiled for 10 min, then identified by western blotting with an antibody against Pan-pY. To identify the phosphorylation sites of ACAP4, His-tagged ACAP4^CT^ was separated by SDS-PAGE after incubation with Flag-JAK1. The ACAP4 band was digested with gel trypsin. The peptides were analyzed by LC-MS/MS (AB, SCIE triple TOF 5600+) as previously described 16.

### Molecular docking and dynamics simulation

The 3-dimensional structure of the human JAK1 kinase was obtained from the protein data bank with PDB ID 6N7A. The obtained structure was co-crystallized with a potent inhibitor (compound 39), which was used to map out the active site of the enzyme 58. The 3D structure of the protein-ligand complex was prepared using the Protein Preparation Wizard of the Schrodinger suite. We docked the optimized 3D structure of bufalin against the ligand-binding pocket of the already prepared 3D crystal structure of JAK1 kinase. We further carried out different sets of 100 nanosecond molecular dynamics simulations on both the apo (unbound) and ligand-bound JAK1 kinases, followed by post-simulation analyses. The molecular dynamics simulations were conducted using GROMACS 2020.3. All simulation graphical outputs were generated with the XMGRACE software.

### The cellular thermal shift assay (CETSA)

The CETSA was performed as described previously 59. MHCC97H cells plated in 10 cm culture dishes were digested with trypsin. Cell pellets were collected and re-suspended in PBS containing a protease inhibitor cocktail. The cell suspensions were freeze-thawed four times between liquid nitrogen and a 25 °C water bath. After centrifugation at 20000 × g for 20 min at 4 °C, the cell lysates were divided into 2 aliquots, one treated with bufalin (1 μM), and one treated with DMSO for 30 min at room temperature. The respective lysates were divided into smaller (100 µL) aliquots and heated at gradient temperatures (40 °C, 43 °C, 46 °C, 49 °C, 52 °C, and 55 °C) for 5 min, followed by cooling for 5 min in a 25 °C water bath. Then, the lysates were centrifugated at 20000 × g for 20 min at 4 °C, and the supernatants were transferred to new tubes with SDS-PAGE sample buffer added. After the samples were boiled for 10 min at 70 °C, Western blotting was used to analyze the signals.

### Immunofluorescence

MHCC97H cells were transfected with ARF6-mCherry plasmids for 24 h, then stimulated with IL6 (50 ng/mL) for 20 min. Cells were fixed in 3.7% paraformaldehyde for 10 min and then permeabilized by 0.2% Triton X-100 in PBS for 3 min. After anti-ACAP4 antibody (PA5-82729, Thermo Fisher) incubation and DAPI staining, the images were visualized using the DeltaVision deconvolution microscope. MHCC97H cells were stimulated with IL6 (50 ng/mL) for 20 min. After being fixed, permeabilized, and blocked, the cells on coverslips were incubated with antibodies against p-ACAP4^Y843^ (Hangzhou HuaAn Biotechnology Company, China), ACAP4 (PA5-82729, Thermo Fisher), or JAK1 (66466-1-Ig, Proteintech) for overnight at 4 °C. The second antibody was incubated with cells for 1 h at room temperature. After phalloidin and DAPI staining, the images were visualized by the DeltaVision deconvolution microscope.

### Wound healing and Transwell migration assay

Confluent MHCC97H cells, MHCC97H cells with ACAP4 KO, and MHCC97H cells with ACAP4 KO expressed exogenous ACAP4^WT^, ACAP4^Y843F^, ACAP4^Y843E^ in a 6-well plate, which was scratched with a 10 μL pipette tip. Then, IL6 (50 ng/mL) was added for 12 h at 37°C. The images were visualized by the 4 × objective of an inverted fluorescence microscope (MF53, China). The scratch area was analyzed by Image J software (National Institutes of Health, USA) and the result was presented in percent wound closure. (The percent wound closure= ((0 h area - 6/12 h area)/0 h area) ×100%).

Besides, MHCC97H and HepG2 cells were collected and resuspended in a serum-free medium. Cells were then plated on the upper chamber of the transwell system (Corning, New York, USA). Cell growth medium was added in the chambers of 24-well plates. With or without IL6 stimulation, bufalin or itacitinib treatment for 24 h, cells were fixed with 4% paraformaldehyde and stained with 0.1% crystal violet staining solution (E607309, Sangon, Shanghai, China). Stained cells were visualized and recorded under a Echo Revolve Fluorescence Touchscreen Microscope (RVL2-K, USA).

### Immunohistochemistry

The fixed, dehydrated, and embedded tissues were cut into sections at a thickness of 4 μm. The paraffin slices were baked in an oven at 65°C for 2 h and washed with PBS three times. Then, the slices were repaired by microwave in EDTA buffer and put into a 3% hydrogen peroxide solution for 10 min at room temperature to block endogenous peroxidase. The indicated antibodies were used to incubate specimens overnight at 4 ℃. 100 μL freshly prepared DAB solution was added to each slice. The pictures were captured by a fluorescence microscope (Nikon Eclipse Ti-SR, Japan). Cells stained with the indicated antibody were analyzed per field at 400 ×magnification. The expression levels of p-ACAP4^Y843^, ACAP4 (PA5-31645, Thermo Fisher), p-JAK1 (#74129, CST), and JAK1 (66466-1-Ig, Proteintech) were scored semiquantitatively based on the histochemistry score (H-Score) as described elsewhere 60. HistoScore = ((1×% weakly stained cells) + (2×% moderately stained cells) +(3×% strongly stained cells)).

### HCC Orthotopic Xenograft Mice Model

Male BALB/c nude mice (n=12, 4-5 weeks) were purchased from SPF (Beijing) Biotechnology Co., Ltd and bred at the Animal Experiment Center of Beijing University of Chinese Medicine, and all procedures were approved by the Animal Care and Use Committee of Beijing University of Chinese Medicine. Under pentobarbital sodium anesthesia (50 mg/kg, i.p.), a surgical incision of approximately 0.8 cm in width was made between xiphoid process and lower margin of liver to expose the right lobe of the liver. MHCC97H-luciferase cells (2× 10^6^), in a total volume of 0.05 mL of DMEM-cell mixture, were injected into the incision to establish the orthotopic xenograft nude mouse models of HCC. Obvious tumor masses and fluorescence appeared in the abdomen of the mice at 2 weeks after surgery. The HCC orthotopic nude mice (n=10, 2 mice died after surgery) were randomly divided into two groups. Mice in model group received intraperitoneal injection of bufalin (Shanghai yuanye Bio-Technology Co., Ltd, #465-21-4, 500 μg/kg, dissolved in 10%DMSO and 90%corn oil) for 2 weeks. Mice in control group were received intraperitoneal injections of a solution consisting of 10% DMSO and 90% corn oil for a duration of 2 weeks. The HCC tumor that express luciferase were analyzed by MIIS Imaging System (Molecular Devices).

### Statistical analysis

All experimental data were statistically analyzed and plotted by GraphPad Prism 5 (GraphPad software). Measurement data were expressed as the mean ± standard error (SEM). Student unpaired t-test was used to compare the mean of two samples, and One-Way ANOVA was used to compare the mean of multiple samples. When variance analysis results were statistically significant, the Tukey test was used for post-hoc comparisons between any two groups. Each experiment was independently repeated three times, and *p*<0.05 was considered statistically significant.

## Supplementary Material

Supplementary figures and tables.
