# Supplementary material for: Kupffer Cell-derived IL6 Promotes Hepatocellular Carcinoma Metastasis Via the JAK1-ACAP4 Pathway
Source: Int J Biol Sci. 2025 Jan 1;21(1):285–305. doi: 10.7150/ijbs.97109 (PMC11667824; doi:10.7150/ijbs.97109)
Supplement: Supplementary file 1 — Supplementary figures and tables. [file ijbsv21p0285s1.pdf]

Supplementary Materials for

Kupffer Cell-derived IL6 Promotes Hepatocellular Carcinoma Metastasis Via the JAK1-ACAP4 Pathway

This file includes:

Figure S 1 to 10

Table S 1, 2a and 2b

Materials and Methods

Reagents

The standard substance of active ingredients of natural products and traditional Chinese medicine (TCM) were purchased from Chengdu Herbpurify co., Ltd (China) and Shanghai yuanye Bio-Technology Co., Ltd (China). The antibodies against CD81 (ab109201), and TSG101 (ab125011) was purchased from Abcam (USA). The antibodies against HSP70 (4872S), CALNEXIN (2679T), p-JAK1 (#74129), and p-STAT3 (#9145) were purchased from Cell Signaling Technology (USA). The antibodies against ARF6 (MA5-11795), JAK1 (66466-1-Ig), STAT3 (60199-1-Ig), and ACAP4 (PA5-31645, PA5-82729), were purchased from Thermo Fisher (USA). The antibody against p-JAK1 (SAB4300123) was purchased from Sigma (USA). The IL6 ELISA kit was purchased from Cloud-Clone Corp (China). The CCK8 kit (MA0218-5) was purchased from Meilun Biotech Co., Ltd (China). The PKH26 red fluorescent cell linker mini kit (#MKCM8734) was purchased from Sigma-Aldrich (USA).

Methods

Cytotoxicity test of MHCC97H cells treated with active components of natural products and traditional Chinese medicine by CCK8 assay

MHCC97H cells were inoculated into a 96-well plate with 100 μL cell suspension per well, with 3 repeats in each group. After incubated at 37 °C overnight, cell culture medium was replaced by complete culture medium containing active ingredients with concentrations of 0.01 μM, 0.1 μM, 1 μM, 10 μM, and 100 μM for 24 h. Then, culture medium was discarded and CCK8 detection solution containing 90 μL DMEM and 10 μL CCK8 was added to each well to culture in 37 °C for 30 min. The absorbance value at 450 nm wavelength was detected by SpectraMax® iD5 Multi-Mode Microplate Readers. The cell survival rate was used to evaluate the cytotoxicity of active ingredients.

Exosomes identification by transmission electron microscope and nanoparticle

tracking analysis

The morphological characteristics of exosomes were analyzed by transmission electron microscope and nanoparticle tracking analysis. Take 5 μL of exosomes dissolved in PBS, drop it on the copper net and incubate at room temperature for 5 min. Absorb the excess liquid from one side of the copper net, add a drop of 2% uranyl acetate to the copper net and incubate at room temperature for 1 min. Then, dry the excess liquid from one side of the copper net with absorbent paper. After dry at room temperature for 20 min, the morphology of exosomes was captured by Tecnai G2 Spirit BioTwin (FEI, USA). The isolated exosomes were thawed on ice, and diluted with 1×PBS. ZetaVIEW S/N17-310 (Particle Metrix, Germany) was used for particle size analysis.

Exosomes labeled by PKH26

The exosomes were labeled with PKH26 red fluorescent cell linker. 2 μL PKH26 reagent was added to the EP tube containing 500 μL Diluent C and gently mixed. Then, the exosomes (< 25 μL) was added to a new EP tube containing 500 μL Diluent C and mix gently. Mix 2 EP solution gently, incubate 1-5 min avoid light at room temperature. 1mL serum was added to EP tube for neutralization. The above solution and normal culture medium was added to the cell petri dish at ratio of 1/4 (vol/vol). After co-culture with the cells for 2 h, the cells were washed 3 times with PBS, fixed with 3.7% paraformaldehyde at 10 min, After DAPI staining, the cells were visualized by DeltaVision deconvolution microscope.

Screening of natural products ingredients against HCC for targeting JAK1

In this study, literature related to clinical studies on natural products and TCM treatment of liver cancer was retrieved. Web of science and Pubmed databases were selected for English database retrieval. Chinese databases were selected including CNKI Database (CNKI), VIP Chinese Science and Technology Full-text Database (VIP) and Wanfang Chinese Database (WANFANG). By utilizing the TCM Inheritance Inheritance Support System (TCMISS), we built the database about natural products and TCM against HCC. Then, we screened the top active ingredients for further investigation.

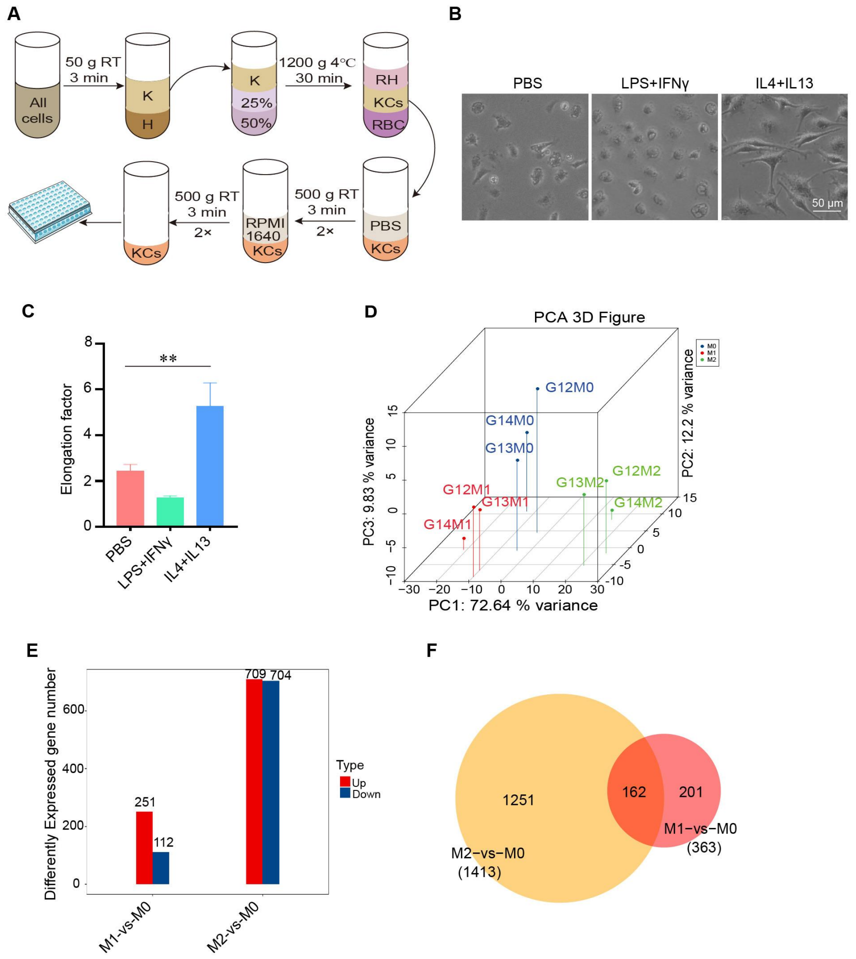

Figure S1 Isolation and polarization induction of Kupffer cells.

(A) Procedure of Isolation of mouse liver kupffer cells (KCs) by percoll density gradient centrifugation.

(B) The KCs morphology were changed after induction with LPS+IFN-γ and IL4+IL13 under light microscope.

(C) The morphological changes were analyzed by calculating the ratio of the cell's long axis to its short axis. All experiments were performed on three independent occasions, and the data are presented as the average ± SEM. $^{**}p < 0.01$.

(D) Principal component analysis (PCA) showed good repeatability among the three samples.

(E) Histogram of differentially expressed genes of M1 and M2-type macrophages compared with M0-type macrophages.

(F) Venn diagram plots showing the overlapping of M0 and M1/2-type macrophages-correlated genes.

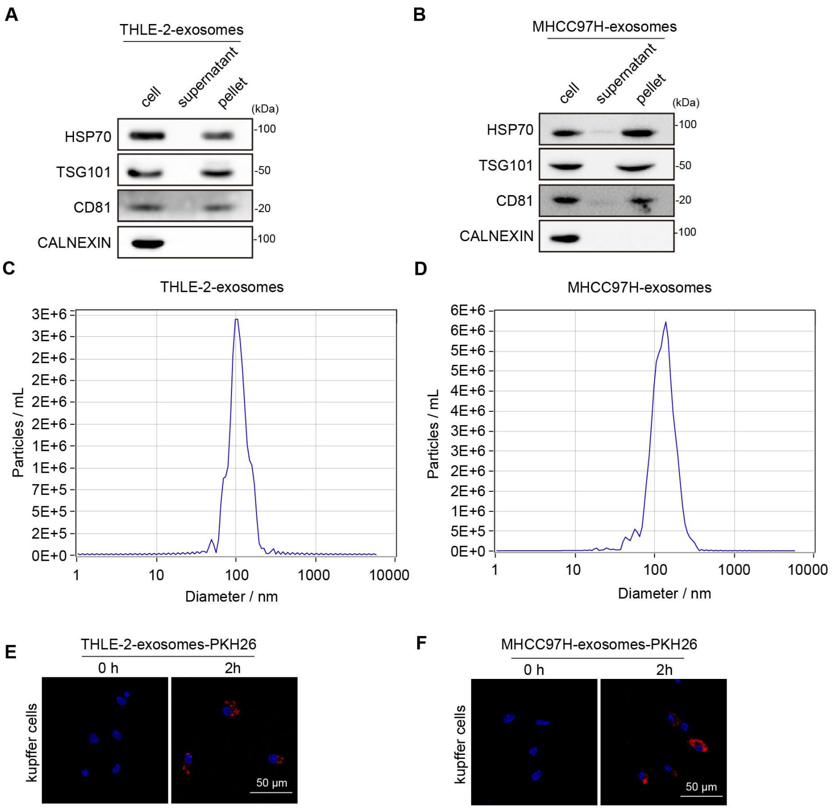

Figure S2 Identification and uptake of exosomes.

(A, B) Western blotting identified the exosomes markers derived from THLE-2 and MHCC97H cells.

(C, D) Particle size analysis of exosomes derived from THLE-2 and MHCC97H cells.

(E, F) PKH26 labeled exosomes derived from THLE-2 and MHCC97H cells were taken up by KCs, the images were acquired under a Delta Vision microscope (scale bar, 50 μm).

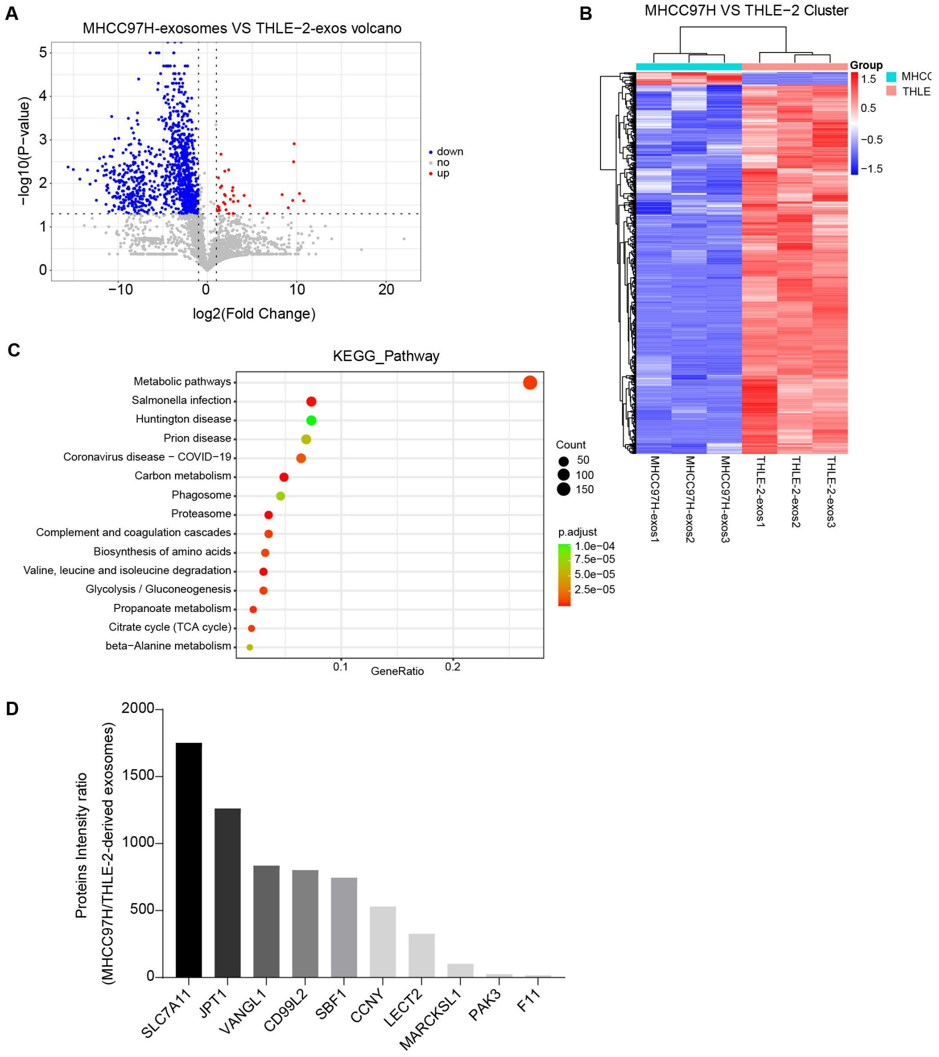

Figure S3 Proteomic analysis of exosomes derived from THLE-2 and MHCC97H cell lines.

(**A**)Volcanic plot analysis of proteins isolated from exosomes of THLE-2 and MHCC97H cell lines.

(**B**)Hierarchical cluster analysis of proteins isolated from exosomes of THLE-2 and MHCC97H cell lines.

(**C**)Analysis of KEGG pathways associated with differentially expressed proteins isolated from exosomes of THLE-2 and MHCC97H cell lines.

(**D**)The ten highest intensity ratios of proteins isolated from exosomes derived from MHCC97H and THLE-2 cell lines.

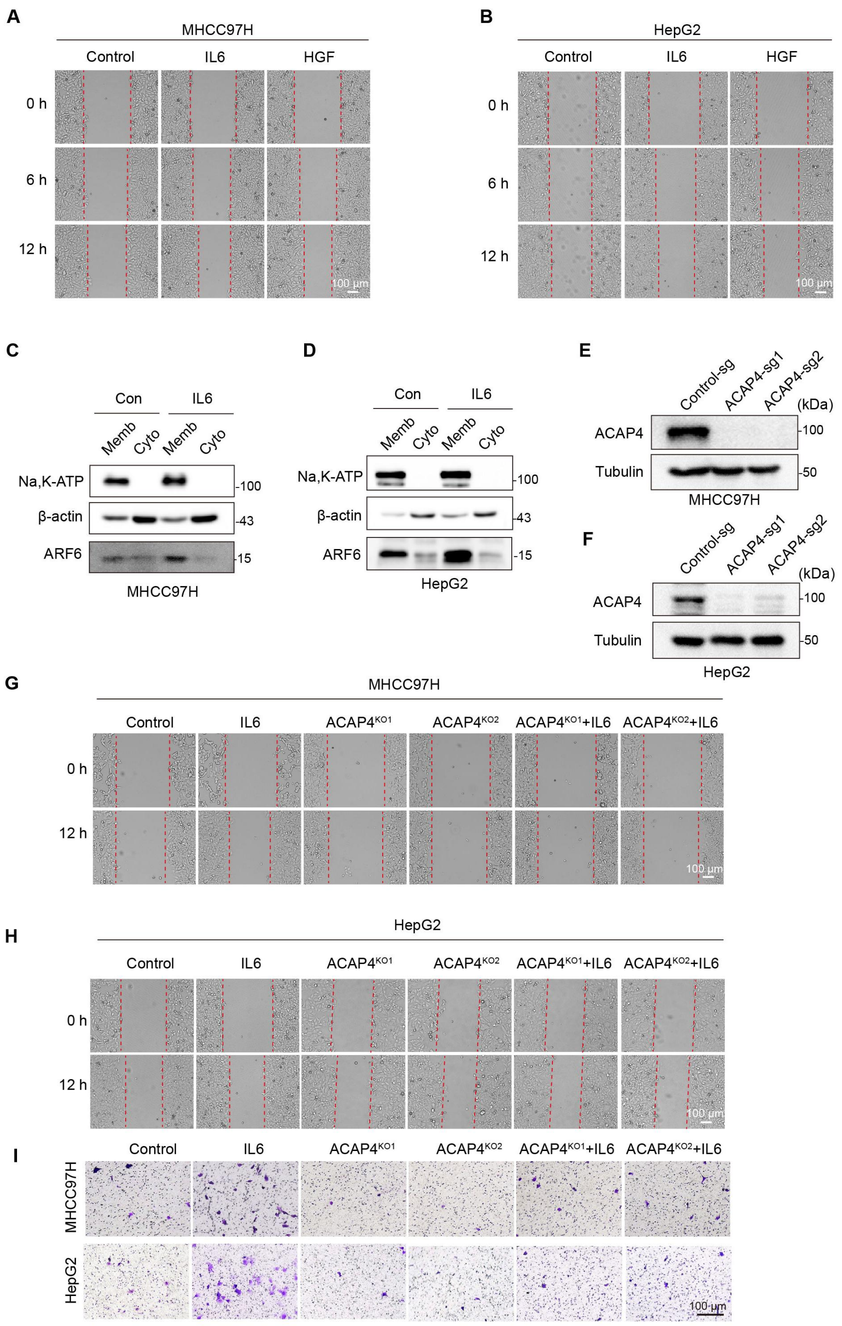

Figure S4 ACAP4 is necessary for IL6-induced HCC cell migration.

(A, B) Wound healing assay of MHCC97H and HepG2 cells stimulated by IL6 (50 ng/mL) for 6 -12 h (scale bar, 100 μm).

(C, D) Western blot analysis was conducted to assess the levels of ARF6 protein in both cytoplasmic and membrane fractions, under conditions with and without IL6 stimulation.

(**E, F**)MHCC97H and HepG2 cells were transfected with ACAP4-sgRNA for 36 h, and then, puromycin (2 μg/mL) was added for 3 days to screen out cells with puromycin resistance. The knockout (KO) effect of ACAP4 was detected by western blotting.

(**G, H**)Wound healing assay of MHCC97H and HepG2 cells and ACAP4 KO ($ACAP4^{KO}$) MHCC97H and HepG2 cells stimulated by IL6 (50 ng/mL) for 12 h (scale bar, 100 μm).

(I) Transwell migration assay of MHCC97H and HepG2 cells and $ACAP4^{KO}$ MHCC97H and HepG2 cells stimulated by IL6 (scale bar, 100 μm).

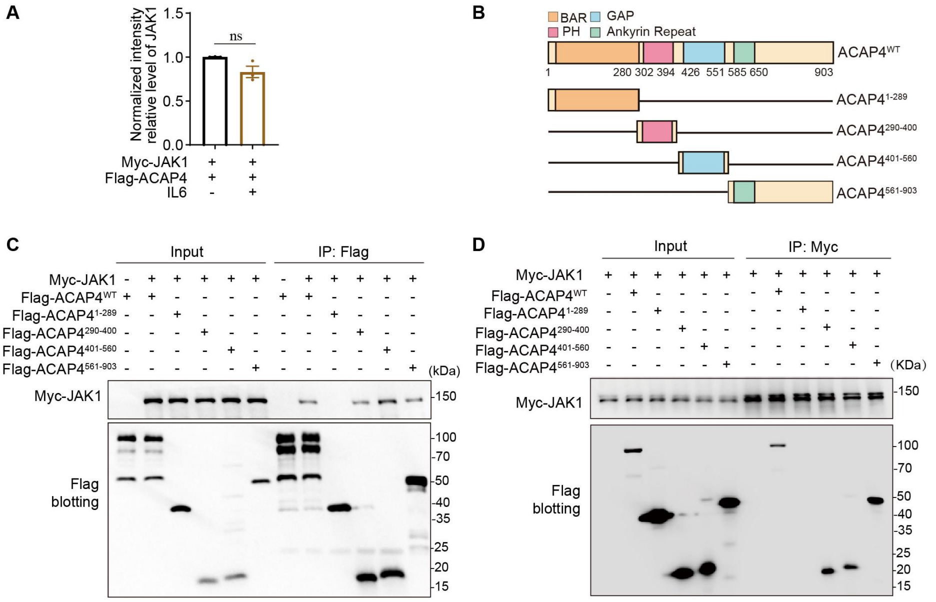

Figure S5 Co-immunoprecipitation of JAK1 with ACAP4 with or without IL6 stimulation and schematic diagram of ACAP4 protein structure features.

(**A**)Quantitative analysis was conducted to assess the Myc-JAK1 protein levels in MHCC97H cells that were co-transfected with Flag-ACAP4 and Myc-JAK1, followed by immunoprecipitation using anti-Flag beads, with or without IL-6 (50 ng/mL) stimulation. The data are presented as the average ± SEM. $^{ns}p > 0.05$.

(B) Schematic diagram of ACAP4 protein structure features and its different truncations with features including ACAP4$^{1\text{-}289}$, ACAP4$^{290\text{-}400}$, ACAP4$^{401\text{-}560}$, and ACAP4$^{561\text{-}903}$.

(C) Flag-ACAP4-FL (Flag-ACAP4^{WT}) expressed in 293T cells alone and Flag-ACAP4-FL, Flag-ACAP4$^{1\text{-}289}$, Flag-ACAP4$^{290\text{-}400}$, Flag-ACAP4$^{401\text{-}560}$, and Flag-ACAP4$^{561\text{-}903}$ truncations co-transfected with Myc-JAK1. Immunoprecipitation was conducted utilizing anti-Flag beads, and the Myc-JAK1 protein was subsequently analyzed through western blotting employing a anti-Myc antibody.

(**D**) Myc-JAK1 expressed in 293T cells alone and Myc-JAK1 with Flag-ACAP4^{WT}, Flag-ACAP4$^{1\text{-}289}$, Flag-ACAP4$^{290\text{-}400}$, Flag-ACAP4$^{401\text{-}560}$, and Flag-ACAP4$^{561\text{-}903}$ truncations co-transfection. Immunoprecipitation was conducted utilizing anti-Myc beads, and the Flag-ACAP4 truncations protein were subsequently analyzed through western blotting employing a anti-Flag antibody.

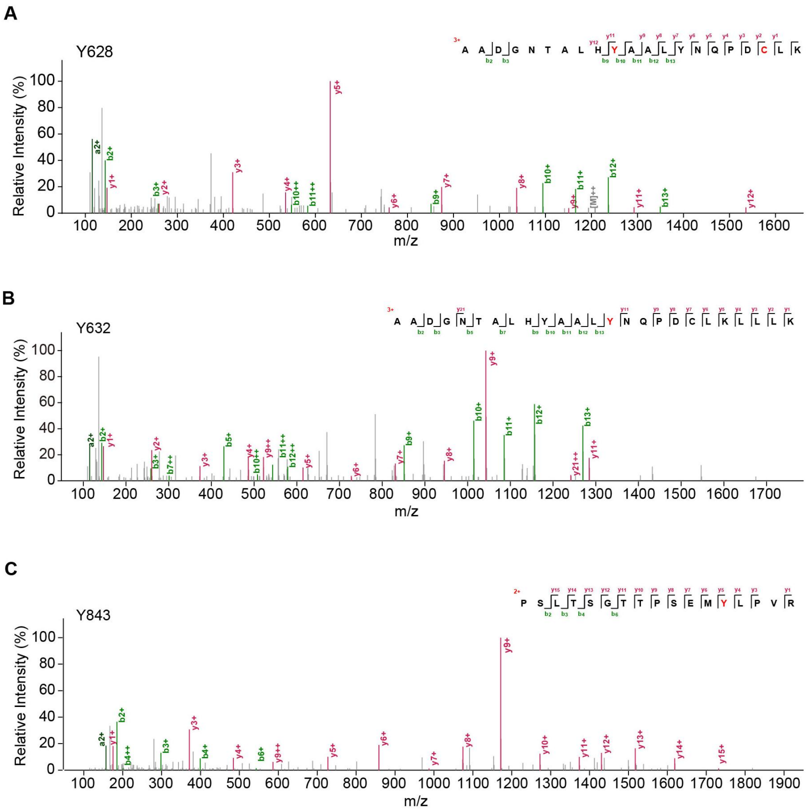

Figure S6 MS/MS spectra of tryptic peptides of purified recombinant ACAP4 protein after *in vitro* phosphorylation.

(A) Representative mass spectrum of phosphorylated Tyr 628 detected in purified ACAP4 protein after *in vitro* phosphorylation.

(B) Representative mass spectrum of phosphorylated Tyr 632 detected in purified ACAP4 protein after *in vitro* phosphorylation.

(C) Representative mass spectrum of phosphorylated Tyr 843 detected in purified ACAP4 protein after *in vitro* phosphorylation.

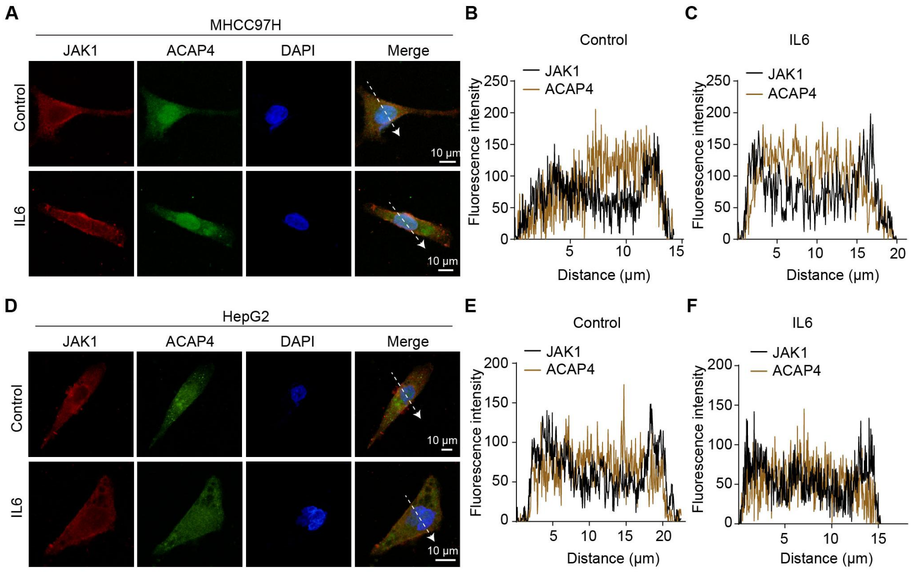

Figure S7 Immunofluorescence staining of the colocalization of JAK1 and ACAP4 in MHCC97H and HepG2 cells, both in the presence and absence of IL6 stimulation.

(**A**)Immunofluorescence staining was employed to assess the colocalization of JAK1 and ACAP4 in MHCC97H cells, both in the presence and absence of IL6 stimulation (scale bar, 10 μm).

(**B-C**)Quantitative analysis of the fluorescence intensity of JAK1 and ACAP4 in the control and IL6-treated groups corresponding to the dashed line in the fluorescence images of MHCC97H cells.

(**D**)Immunofluorescence staining was employed to assess the colocalization of JAK1 and ACAP4 in HepG2 cells, both in the presence and absence of IL6 stimulation (scale bar, 10 μm).

(**E-F**)Quantitative analysis of the fluorescence intensity of JAK1 and ACAP4 in the control and IL6-treated groups corresponding to the dashed line in the fluorescence images of HepG2 cells.

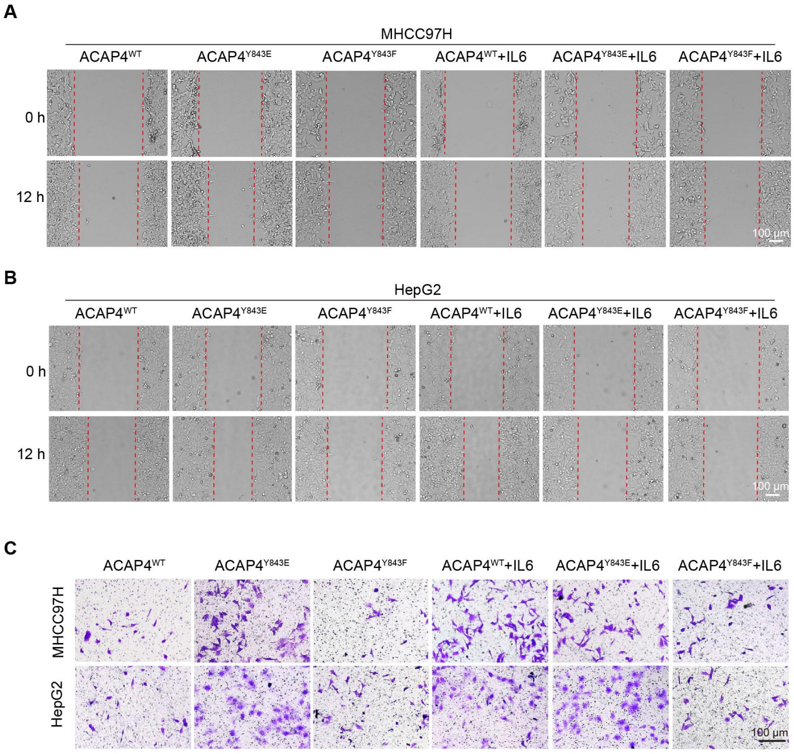

Figure S8 Migration assay of ACAP4^{KO} MHCC97H and HepG2 cells expressing exogenous ACAP4^{WT}, ACAP4^{Y843E}, or ACAP4^{Y843F} with or without IL6 stimulation.

(**A, B**) Wound healing assay of ACAP4^{KO} MHCC97H and HepG2 cells expressing exogenous ACAP4^{WT}, ACAP4^{Y843E}, or ACAP4^{Y843F} with or without IL6 stimulation (scale bar, 100 μm).

(**C**)Transwell migration assay of ACAP4^{KO} MHCC97H and HepG2 cells expressing exogenous ACAP4^{WT}, ACAP4^{Y843E}, or ACAP4^{Y843F} with or without IL6 stimulation (scale bar, 100 μm).

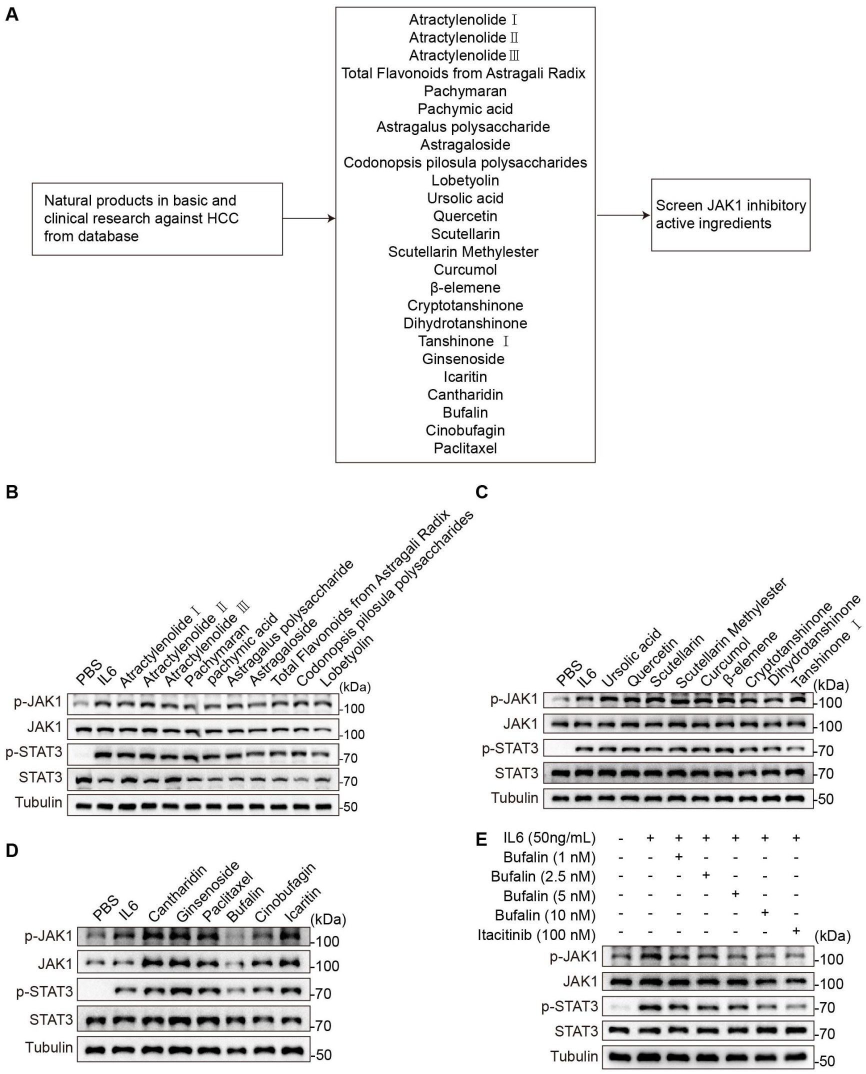

Figure S9 Screening of natural products and traditional Chinese medicine derived active ingredients against HCC for targeting JAK1.

(A) The screening process of natural products and TCM against HCC and the top active ingredients were selected for further verification.

(B-D) Western blotting analysis was conducted to assess the expression levels of p-JAK1, JAK1, p-STAT3, and STAT3 in MHCC97H cells following treatment with various active ingredients and subsequent stimulation with IL6.

(E) Western blotting analysis was conducted to assess the expression levels of p-JAK1, JAK1, p-STAT3, and STAT3 in MHCC97H cells following treatment with different concentrations of bufalin and subsequent stimulation with IL6.

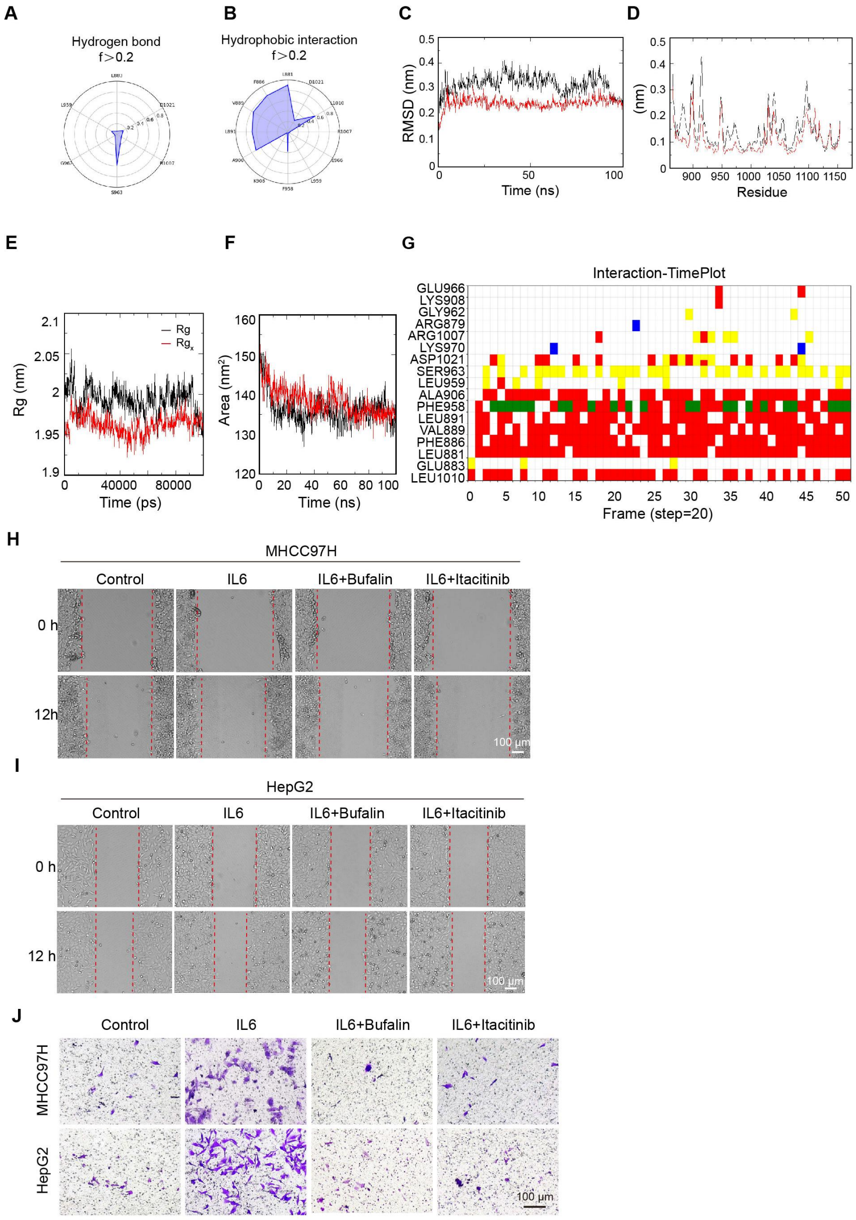

Figure S10 Bufalin interacted with JAK1 kinase and inhibited the cell migration.

(A, B) The characteristics of hydrogen bonding and hydrophobic dynamic interactions related to bufalin, respectively.

(C) The RMSD plot of the JAK1 kinase and its complex with bufalin.

(D) The RMSF plot of the JAK1 kinase and its complex with bufalin.

(E) The Rg plot of the JAK1 kinase and its complex with bufalin.

(F) The SASA plot. The trajectories representing the apo protein (JAK1 kinase) and its complex with bufalin are shown in black and red colors, respectively.

(G) Interaction time plot from the 100 nanosecond protein-ligand molecular dynamics simulation summarized into 20 frames. The red, yellow, green, and blue colors denote

the hydrophobic, hydrogen bond, pi stacking, and salt bridge interactions, respectively.

(H, I) Wound healing assays were conducted on MHCC97H and HepG2 cells, which were pretreated with bufalin or itacitinib and subsequently stimulated with IL6 at a concentration of 50 ng/mL (scale bar, 100 μm).

(**J**)Transwell migration assays were performed on MHCC97H and HepG2 cell lines that had been pretreated with PBS, bufalin, or itacitinib, with all treatments conducted in the presence of IL6 stimulation (scale bar, 100 μm).

Table S1 Clinicopathological characteristics of hepatocellular carcinoma (HCC) patients and normal participants from hospital.

Case No.	Gender	Age	Cancer Subtype	Cancer Stage (BCLC)
#H1	male	47	HCC	A
#H2	male	63	HCC	A
#H3	female	51	HCC	A
#H4	male	63	HCC	A
#H5	male	50	HCC	A
#H6	male	46	HCC	A
#H7	male	64	HCC	A
#H8	male	56	HCC	A
#H9	female	66	HCC	B
#H10	male	56	HCC	B
#H11	male	78	HCC	B
#H12	male	45	HCC	B
#H13	male	66	HCC	B
#H14	male	52	HCC	C
#H15	male	66	HCC	C
#H16	male	64	HCC	C
#H17	female	53	HCC	C
#H18	male	64	HCC	C
#H19	male	54	HCC	C
#H20	male	58	HCC	C
#N1	Male	58	NO	NO
#N2	Male	59	NO	NO
#N3	Male	67	NO	NO
#N4	Female	57	NO	NO
#N5	Male	63	NO	NO
#N6	Male	64	NO	NO
#N7	Male	64	NO	NO
#N8	Female	69	NO	NO
#N9	Female	61	NO	NO
#N10	Female	55	NO	NO

#H: HCC patients, #N: Normal participants.

Table S2a. Cytotoxicity of different concentrations of active components of traditional Chinese medicine against HCC.

Ingredients/Concentration	0	0.1	1	10	100
AtractylenolideⅠ(μM)	100.0±0. 5	95.5±4.3	94.7±7.0	97.0±7.0	99.2±8.3
AtractylenolideⅡ(μM)	100.1±5. 3	100.7±0.2	106.0±3.0	102.8±1.1	75.3±2.1**
AtractylenolideⅢ(μM)	100.1±5. 3	100.4±3.0	101.3±5.6	99.2±2.7	104.3±6.2
Total Flavonoids from Astragali Radix (μg/ml)	100.0±0. 5	101.2±0.5	97.9±4.0	97.4±4.7	89.2±4.0**
Pachymaran（μM）	99.9±1.6	97.5±2.9	97.0±4.7	95.4±3.9	93.5±6.7
Pachymic acid (μM)	100.0±0.9	97.9±0.2	98.4±2.6	92.7±1.7**	77.7±2.3**
Astragalus polysaccharide (μM)	99.9±1.6	96.5±1.9	94.7±1.3	94.3±1.6	88.6±5.7**
Astragaloside (μM)	100.0±0.9	98.6±3.4	95.9±1.6	100.3±3.4	101.9±3.7
Codonopsis pilosula polysaccharides (μM)	99.9±1.6	94.2±2.2	93.7±2.3	96.8±5.5	97.7±8.1
Lobetyolin (μM)	100.0±0. 5	100.1±2.9	104.9±5.6	99.4±6.6	99.1±9.4
Ursolic acid (μM)	100.0±0. 5	95.3±3.4	101.1±9.2	103.4±5.6	11.3±1.4**
Quercetin (μM)	100.2±6. 5	96.2±5.1	105.1±6.3	102.2±1.0	95.9±9.0
Scutellarin (μM)	100.2±6. 5	98.5±6.6	111.7±2.4	113.3±6.1	106.4±13.5
Scutellarin Methylester (μM)	100.2±6. 5	97.1±0.7	105.4±1.7	103.2±3.8	103.7±3.2
Curcumol (μM)	101.3±1. 1	97.2±3.4	97.7±1.4	98.6±3.8	94.0±3.0*
β-elemene（μg/ml）	100.0±0. 5	99.7±2.2	99.1±0.2	103.3±2.0	101.6±4.3
Cryptotanshinone (μM)	100.0±0. 5	98.6±5.5	103.9±8.9	97.1±2.6	83.3±2.6**
Dihydrotanshinone (μM)	101.3±1. 1	106.5±1.8	105.5±4.4	103.5±6.7	14.2±0.5**
Tanshinone Ⅰ (μM)	101.3±1. 1	104.0±3.0	103.5±2.8	98.4±1.3	41.4±2.0**
Ginsenoside (μM)	100.1±5. 3	97.3±1.1	96.4±3.6	95.2±1.0	100.1±2.7
Icaritin (μM)	101.3±1. 1	101.3±1.9	100.3±0.7	99.5±0.4	27.1±3.6**

Table S2b. Cytotoxicity of different concentrations of active components of traditional Chinese medicine against HCC.

Ingredients/Concentration	0 μM	0.01 μM	0.1 μM	1 μM	10 μM
Cantharidin	100.1±5.3	95.4±3.1	95.3±0.5	82.7±2.9**	25.9±1.2**
Bufalin	100.0±0.9	97.4±2.3	37.1±1.3**	32.3±1.1**	30.9±0.3**
Cinobufagin	100.0±0.9	98.3±1.4	69.1±0.3**	56.4±1.1**	35.6±0.9**
Paclitaxel	100.1±5.3	87.9±3.6	69.1±3.6**	47.9±9.0**	31.5±8.2**

All experiments were performed at three independent occasions, and data are given as the Average ± SEM. *p<0.05, **p<0.01.
